# The Mizoroki–Heck reaction between *in situ* generated alkenes and aryl halides: cross-coupling route to substituted olefins

**DOI:** 10.1039/d3ra03533f

**Published:** 2023-07-25

**Authors:** Anushka Koranne, Shrishty Turakhia, Vikesh Kumar Jha, Sangeeta Gupta, Rangnath Ravi, Abhijeet Mishra, Anil K. Aggarwal, Chandan Kumar Jha, Neelu Dheer, Abadh Kishor Jha

**Affiliations:** a Govt. Shivnath Science College Rajnandgaon 491441 Chhattisgarh India awadhbrave@gmail.com; b Govt. R. R. M. PG College Surajpur Chhattisgarh India; c Shivaji College, University of Delhi Delhi India; d Govt. Engineering College Madhubani Bihar India; e Acharya Narendra Dev College, University of Delhi Delhi India

## Abstract

This review covers palladium-catalyzed typical Mizoroki–Heck cross-coupling reactions of aryl halides with *in situ* generated alkenes, by following a typical Heck coupling mechanism to form substituted olefins unlike direct cross-coupling of alkenes with aryl halides in Heck olefination. These reactions solve the issue of alkenes undergoing polymerization at high temperatures and increase reaction efficiency by reducing the reaction time and purification steps.

## Abstract 2

This review covers palladium-catalyzed typical Mizoroki–Heck cross-coupling reactions of aryl halides with *in situ* generated alkenes, to form substituted olefins unlike direct cross-coupling of alkenes with aryl halides in Heck olefination.

## Graphical abstract

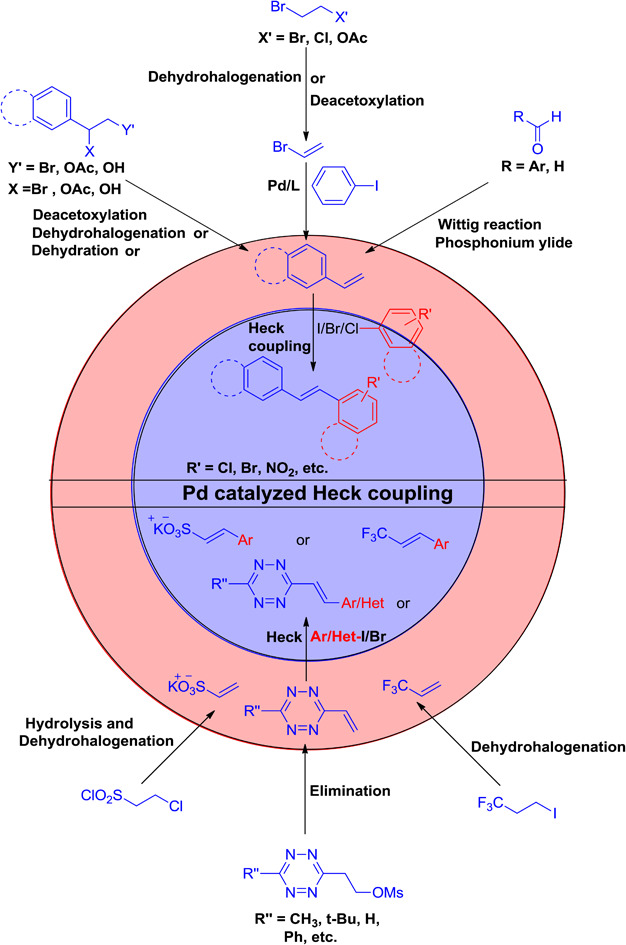

## 1. Introduction

**Scheme 1:**
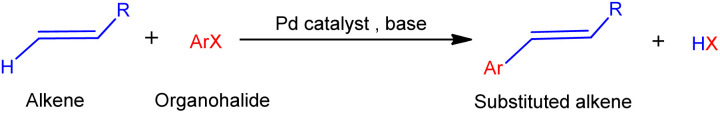
Palladium catalyzed Mizoroki–Heck coupling reaction.

**Fig. 1:**
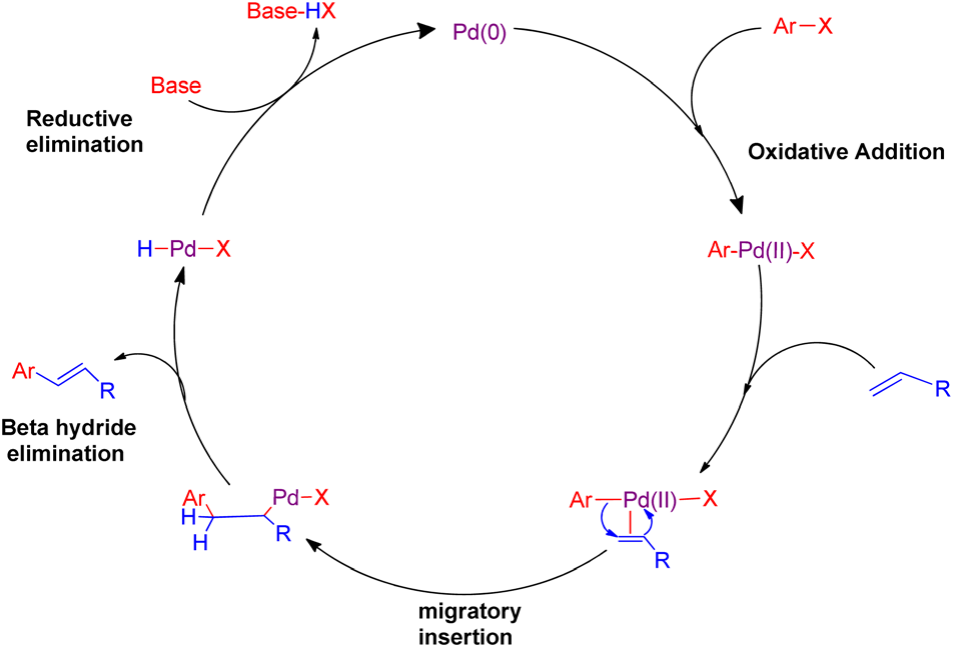
Mechanism of palladium catalyzed Mizoroki–Heck coupling reaction.

Cross-coupling reactions catalyzed by palladium are considered to be the most competent scientific method for the formation of C(sp^2^)–C(sp^2^) bonds.^1^ Most of these reactions proceed through a combination of a nucleophile which is generally an organometallic reagent with an electrophile to provide the corresponding coupling product.^2^ However, one major limitation of this general approach is the necessity of a stoichiometric amount of the organometallic reagents, which are often prepared *via* expensive and multistep procedures. The Heck reaction is considered as the earliest example of a C–C bond forming reaction that does not require a stoichiometric amount of the organometallic coupling partner. It is one of the most interesting reaction catalyzed by palladium, involving the chemical reaction of an unsaturated halide (or triflate) with an alkene in the presence of a base to form a substituted alkene^3,4^ (Scheme 1). The usual mechanism involves mainly four steps which are, oxidative addition, migratory insertion, β-hydride elimination and reductive elimination as shown in Fig. 1. The reaction initiates with oxidative addition of aryl halide to palladium catalyst. Upon introduction of alkene into this, a palladium pie complex gets formed which is then followed by migratory insertion of this alkene into the palladium-aryl bond. Finally β-hydride elimination produces a new substituted olefin. The concluding step of the reaction involves reductive elimination to generate the active palladium catalyst.

This Heck reaction involves various useful transformations which result in the formation of complex organic molecules including bioactive stilbenoids, anticancer agent resveratrol, DMU-212, *etc.*^4^ The reaction has been utilized to create a wide range of natural and synthetic compounds, and in most occasions, the transformation occurs in presence of a suitable aryl halide and styrene or similar olefin, including a small amount of palladium catalyst and a stoichiometric amount of a base.

However, the alkenes necessary for the reaction are either difficult to manufacture or hard to purify due to their tendency to polymerize during distillation or storage. Moreover even on employing commercially available olefins, or synthesizing them *via* various traditional methods available, there remains a possibility of them undergoing polymerization under high reaction temperature conditions and hence are required in higher amount.^5^

**Scheme 2:**
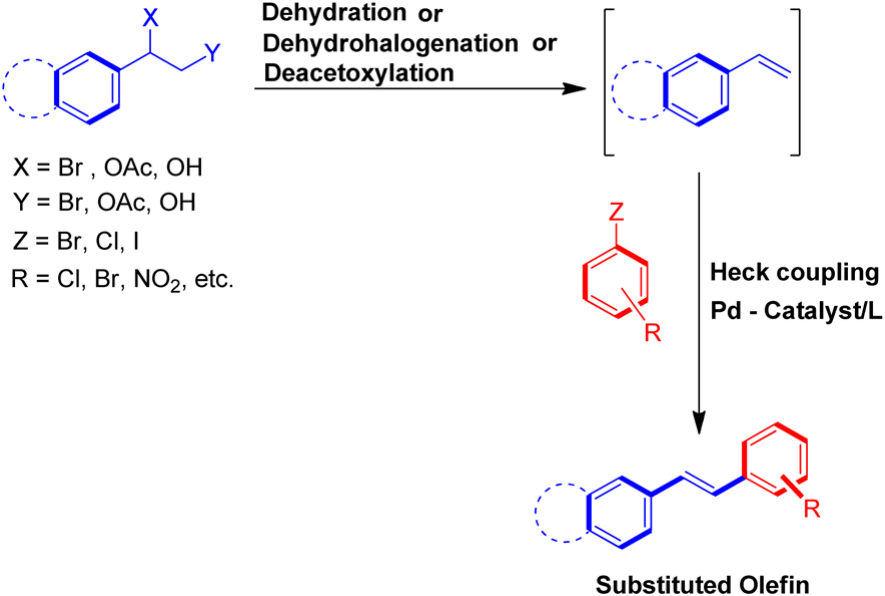
Heck reaction between *in situ* generated alkenes from 1 or 2-bromo/hydroxy/acetoxy alkyl arenes and aryl halides.

**Scheme 3:**
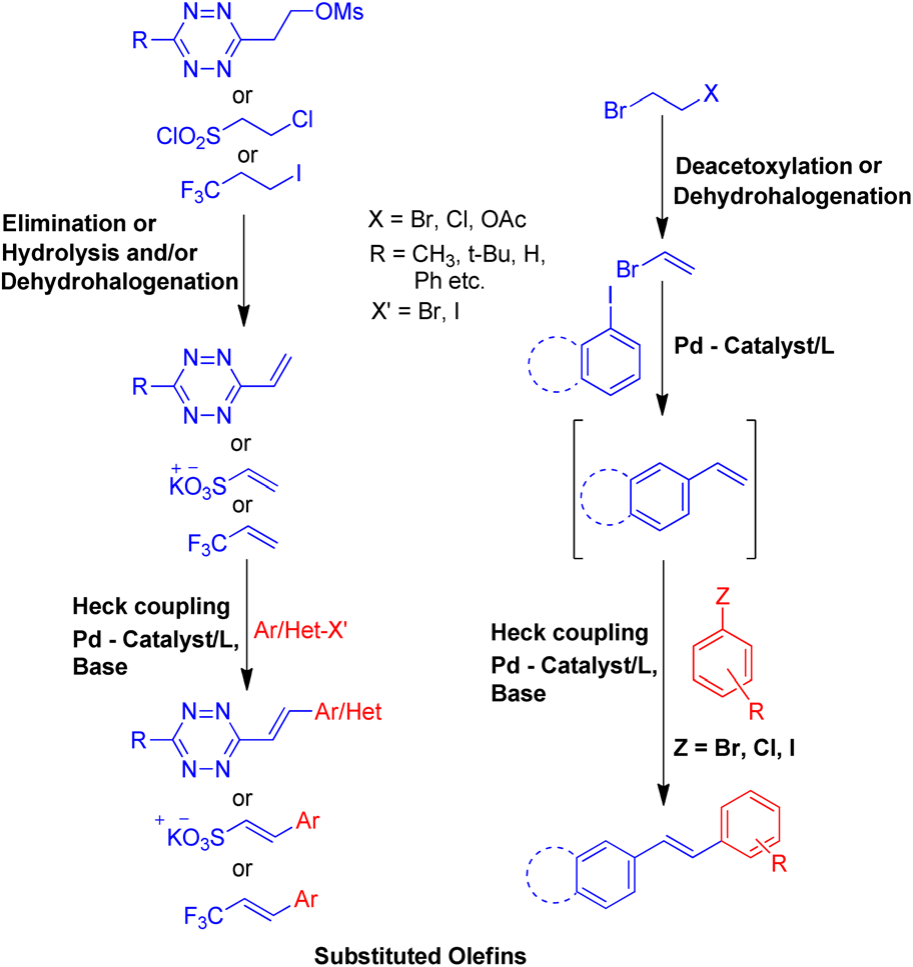
Heck reaction between aryl halide and *in situ* generated alkenes from 2-chloroethanesulfonyl chloride, 1-iodo-3,3,3-trifluoropropane, 1,2,3,4-tetrazine derivatives, 1,2-dihaloethane and 2-halo ethyl acetate with aryl halides.

**Scheme 4:**
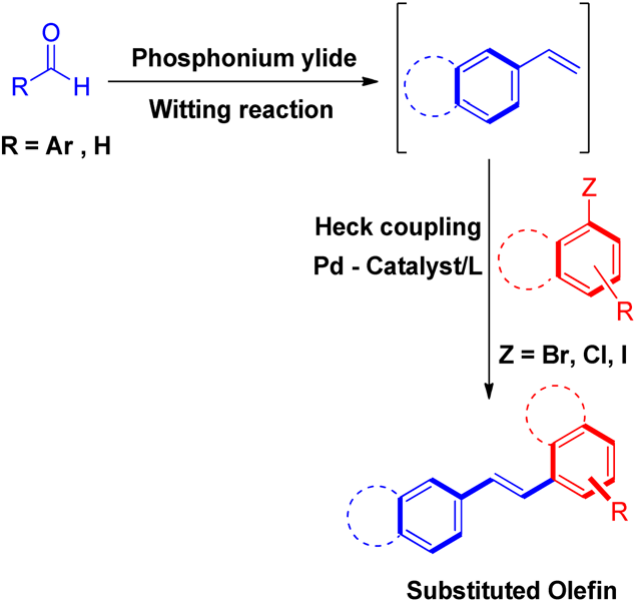
Heck reaction between *in situ* generated Wittig alkenes and aryl halides.

To solve some of these issues, many scientists have reported Heck reaction between *in situ* generated alkenes and aryl halides. The 1st one of these methods include *in situ* generation of required alkenes for Heck coupling from unconventional substrates such as 1 or 2-bromo/hydroxy/acetoxy alkyl arenes (Scheme 2) or 1,2-dihaloethane/2-haloethyl acetate/1,2,3,4-tetrazine derivatives/2-chlorosulfonyl chloride/1-iodo-3,3,3-trifluoropropane (Scheme 3). While the 2nd method utilizes Wittig alkenes for sequential Wittig–Heck process (Scheme 4). In literature there are many reports for the *in situ* synthesis of alkenes by the usage of a carbene precursor such as diazo compounds. However the mechanism when utilizing carbene precursor differs from the traditional Mizoroki–Heck coupling mechanism as it involve a supposed metal-carbene migration process^6^ and is therefore out of the scope of this review. The 1st method as stated above involves *in situ* generation of alkenes *via* various processes such as dehydrohalogenation of 1 or 2-halo ethyl benzene,^5,7^ dehydration of 1 or 2-hydroxy alkyl arene,^7,8^ or deacetoxylation of 1 or 2-acetoxy ethyl benzene (Scheme 2).^7–9^ It also includes the dehydrohalogenation and deacetoxylation of 1,2-dihalo ethane^7^ and 2-haloethyl acetate^7^ respectively, followed by their coupling with aryl halide prior to loosing another functional group. In addition, hydrolysis followed by dehydrohalogenation of 2-chloroethanesulfonyl chloride^10^ or dehydrohalogenation and elimination pathway in 1-iodo-3,3,3-trifluoropropane^11^ and substituted 1,2,3,4-tetrazine^14^ respectively were also some of the methods developed to form the desired substituted olefin (Scheme 3).

The second method of tandem Wittig–Heck reaction^5,15–27^ involves generation of required olefins by reacting aldehyde or ketones with acceptable phosphonium salt and then coupling of these olefin with aryl halide under Heck conditions to result in the formation of desired conjugated species (Scheme 4). Both of these methods of *in situ* generating the suitable olefins and then proceeding further with Heck coupling have been summarized in this review.

## 2. Mizoroki–Heck reaction between *in situ* generated alkenes from 1 or 2-bromo/hydroxy/acetoxy alkyl arenes or 1,2-dihaloethane/2-haloethyl acetate/1,2,3,4-tetrazine derivatives/2-chlorosulfonyl chloride/1-iodo-3,3,3-trifluoropropane and aryl halides

**Scheme 5:**
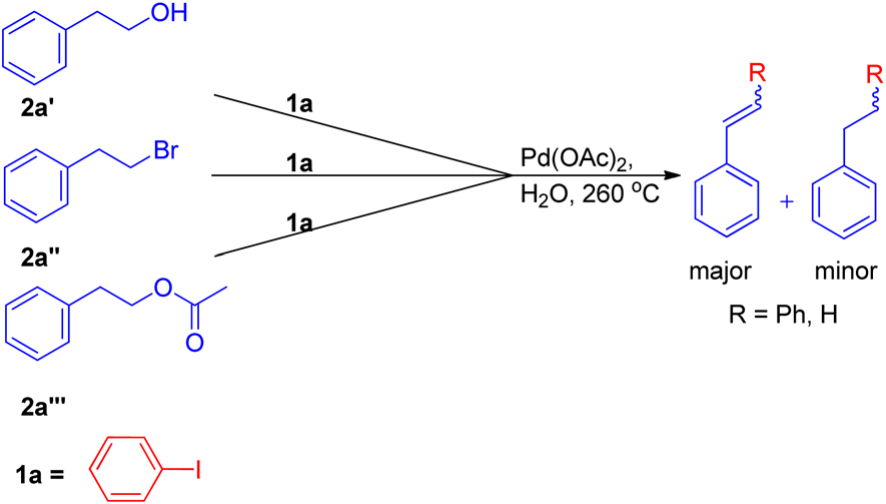
Potential alkene synthons examined in the Heck coupling reaction in superheated water.

The expansion of Heck reaction using more unusual substrates was 1st performed by Edith J. Parsons and co-workers in 1995. Their primary focus was the *in situ* generation of alkenes capable of undergoing coupling.^7^ The usage of “alkene synthons” depends on the unique properties of superheated and supercritical water (SW) which expanded the scope of the Heck reaction to include alkane-based unconventional substrates in addition to the traditional alkene based substrates. They examined a series of alkene synthons in the Heck coupling reaction under supercritical water conditions (Scheme 5). These alkene synthons included, 2-hydroxy ethyl benzene (2a′), 2-bromo ethyl benzene (2a′′), and 2-acetoxy ethyl benzene (2a′′′) (Scheme 5). Apart from them, 1,2-dihaloethane and 2-haloethyl acetate (2aa) were also examined (Scheme 7).

**Scheme 6:**
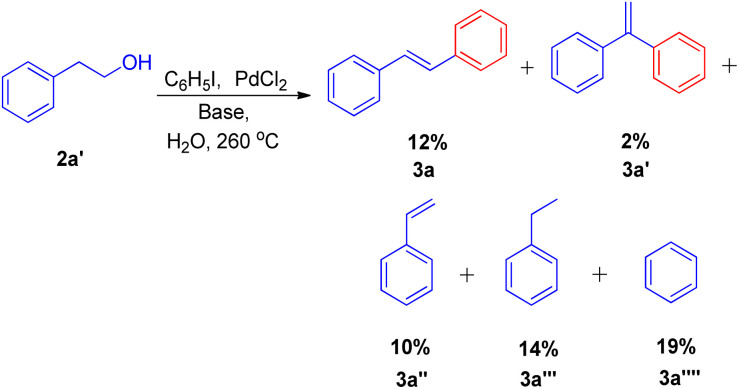
Palladium catalyzed coupling of styrene synthon with iodobenzene.

They substituted styrenes in the coupling reaction with these synthons and made them to react with iodobenzene, using PdCl_2_ or Pd(OAc)_2_ as the catalyst precursor and NaOAc as the base. Each of them were able to form the desired *trans*-stilbene (3a) and some amount of 1,1-diphenylethylene (3a′) (Scheme 6), however the reported yield of the product was slightly lower from what was observed in the reaction of styrene itself. The production of styrene along with its hydrogenated analogue ethylbenzene (Scheme 6; 3a′′ and 3a′′′ respectively) indicated that synthons were defunctionalized under the applied reaction conditions to form alkenes which then underwent coupling with aryl halide. The presence of significant amount of 2-hydroxy ethyl benzene (2a′) was observed in the reaction mixture when 2-acetoxy ethyl benzene (2a′′) and 2-bromo ethyl benzene (2a′′′) were coupled separately with iodobenzene, which suggested that only 2-hydroxy ethyl benzene was responsible for the formation of styrene and the end products. However the effect of presence of various electron rich and electron poor groups on these alkene synthons (2a′, 2a′′, and 2a′′′) is yet to be explored.

**Scheme 7:**
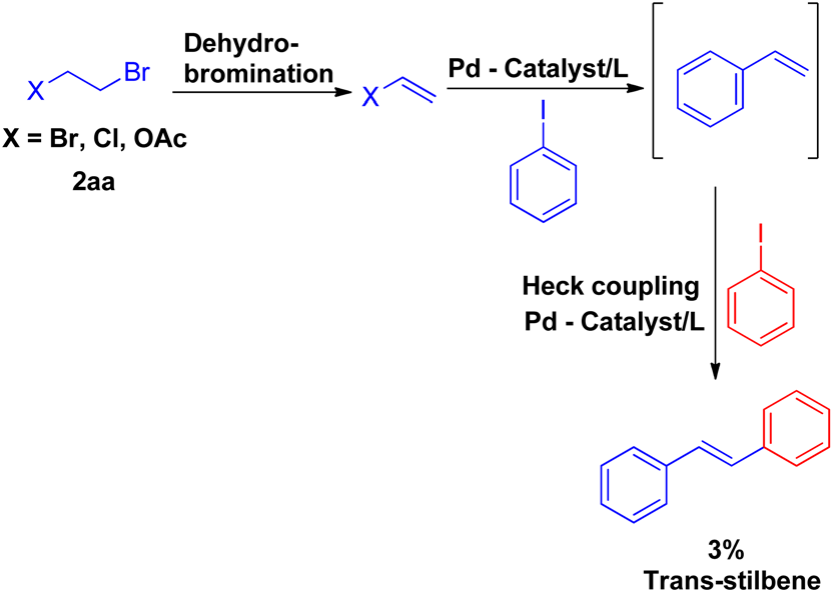
Palladium catalyzed coupling of iodobenzene with alkene generated from 1,2-dihaloethane/2-haloethyl acetate (2aa) and iodobenzene.

Vinyl synthons (2aa) were also examined in superheated water coupling system, and the reaction was found to proceed *via* dehydrobromination first and then coupling with iodobenzene occurred with the loss of other halogen entity to result in styrene which eventually reacted with iodobenzene to form *trans*-stillbene. However the desired stilbene, was obtained in very low yield (Scheme 7).

**Scheme 8:**
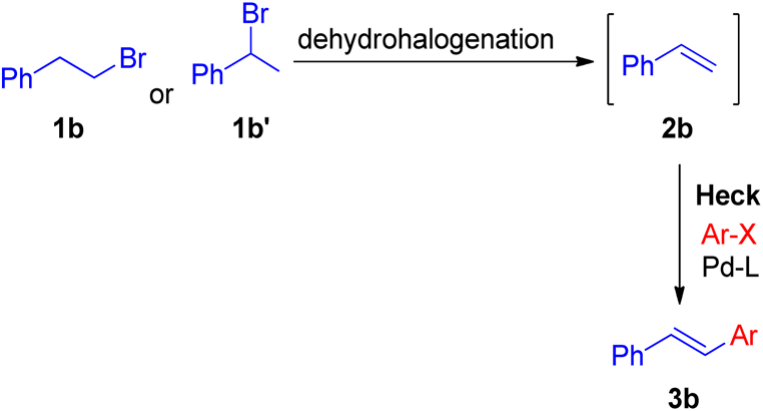
Proposed routes of *in situ* synthesis of styrene for one-pot Heck reaction.

In 2010 Akeel S. Saiyed and Ashutosh V. Bedekar^5^ reported a one pot synthesis which involves olefination followed by the Mizoroki–Heck reaction. The main feature of this method is the *in situ* synthesis of required olefin (2b) from alkyl halides. Olefins were synthesized by base mediated dehydrohalogenation of 1 or 2-bromoalkyl arenes followed by coupling with aryl halide to form stilbene under Mizoroki–Heck condition (Scheme 8).

**Scheme 9:**
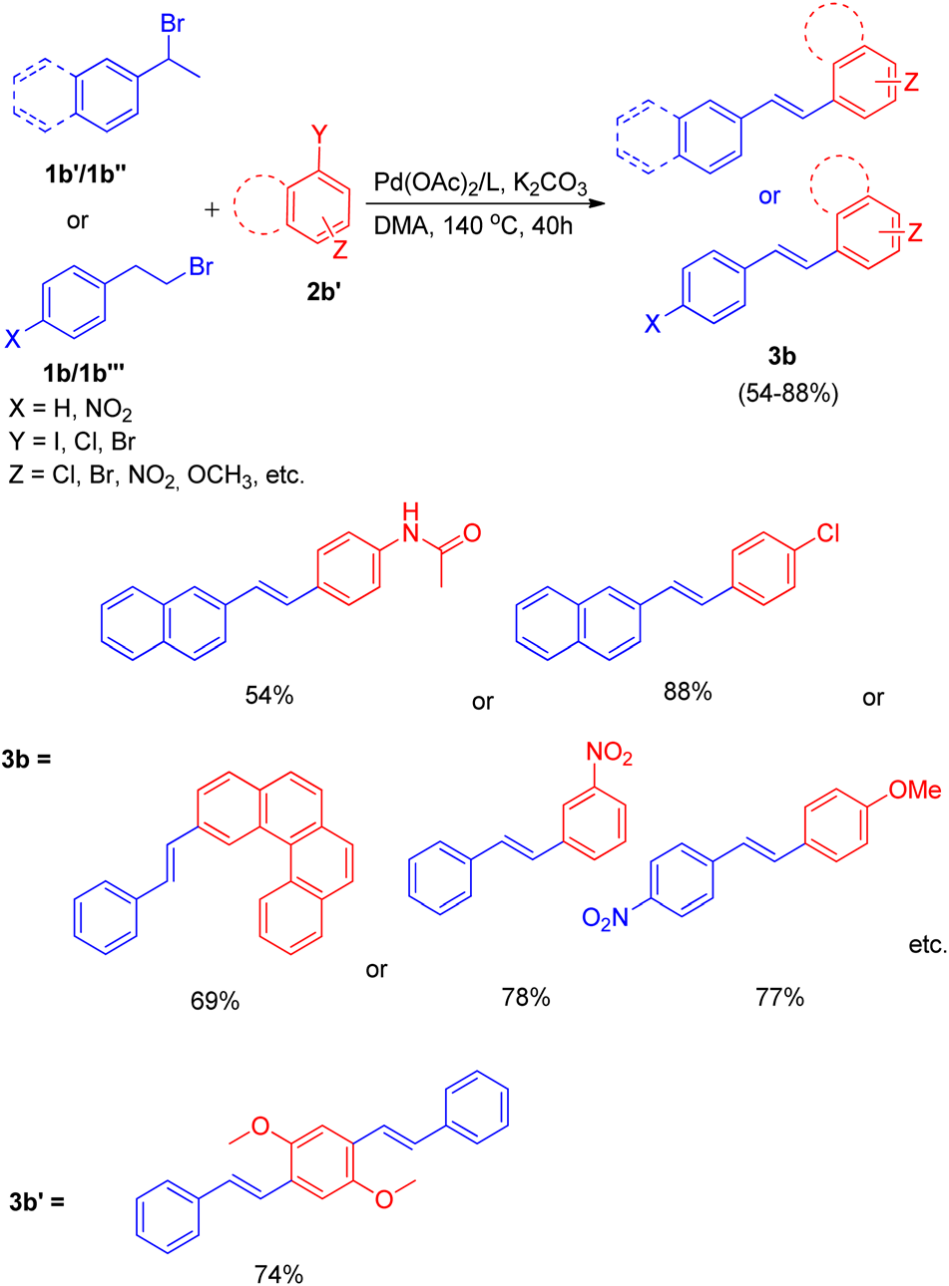
Dehydrohalogenative one-pot Heck reaction.

The optimized conditions (Scheme 9) for this protocol involves reaction between 1.2 equiv. of aryl halide (2b′) and 1 equiv. of alkyl halide (1b–1b′′′) in the presence of Pd(OAc)_2_ (0.5 mol%) as catalyst, 3 equiv. K_2_CO_3_ as base, 0.55 mol% of oxazolinyl ligand in DMA at 140 °C for 40 h to result in formation of substituted olefin in good to excellent yield (54–88%).

**Fig. 2:**
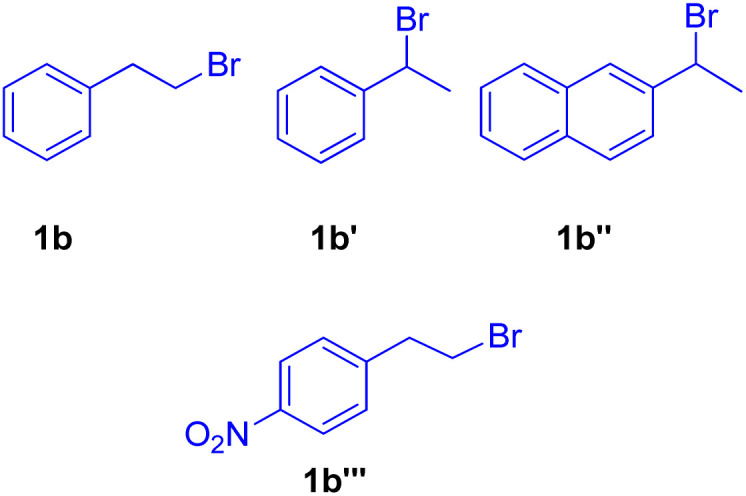
1 or 2-bromoalkyl arenes used for *in situ* generation of olefins by dehydrohalogenation followed by Mizoroki–Heck reaction.

When under the same reaction conditions, 1,4-diiodo-2,5-dimethoxybenzene was used as an aryl halide to undergo coupling with 2-bromoalkyl arene (1b), double dehydrohalogenative Heck reaction was observed which yielded (3b′) the 74% of corresponding stilbene. To show the generality of the dehydrohalogenative Heck approach, a series of 1 or 2-bromoalkyl arenes (Fig. 2) were treated with suitable aryl halide. The reaction mixture containing suitable aryl halide with bromoalkyl arene was made to react under optimized reaction conditions, which resulted in formation of good yield (54–88%) of stilbene derivatives.

**Scheme 10:**
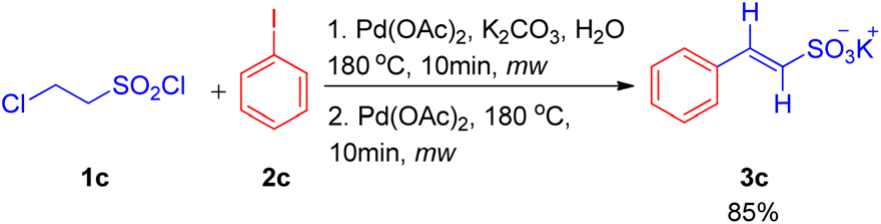
Domino hydrolysis/dehydrohalogenation/Heck coupling pathway for the synthesis of potassium styrene sulfonates.

After the work by Akeel S. Saiyyed and Ashutosh V. Bedekar^5^ on the dehydrohalogenative Heck reaction, another domino hydrolysis/dehydrohalogenation/Heck reaction was reported by G. K. Surya Prakash *et al.* in 2011.^10^ The method employs chloroethanesulfonyl chloride as an alkene synthon which undergoes hydrolysis followed by dehydrohalogenation to form the required olefin for Heck reaction. This alkene then couples with haloarenes to result in the corresponding substituted styrene sulfonate salts. Apart from requiring short reaction time and water as a medium, one of the many advantages offered by this methodology is the utilization of phosphine and additive-free catalytic system. The investigation to attain optimized conditions began by reacting 2-chloroethanesulfonyl chloride (1c) with iodobenzene (2c) in water in the presence of Pd(OAc)_2_ (2 mol%) as catalyst and potassium carbonate as a base at 180 °C for 10 min in the microwave (Scheme 10). Among the analyzed bases such as NaHCO_3_, K_2_CO_3,_ Na_2_CO_3_, and triethylamine, K_2_CO_3_ (2–3 equiv.) resulted in the highest conversion (85%) of the coupling product (3c). Owing to the absence of ligand in the reaction mixture, the possibility of deactivation of Pd(0) catalyst to form palladium black was avoided by the addition of fresh 1 mol% of the catalyst to the previous reaction mixture after heating it for 10 min in microwave (as mentioned above) and heating it again in the microwave at 180 °C for 10 min.

**Scheme 11:**
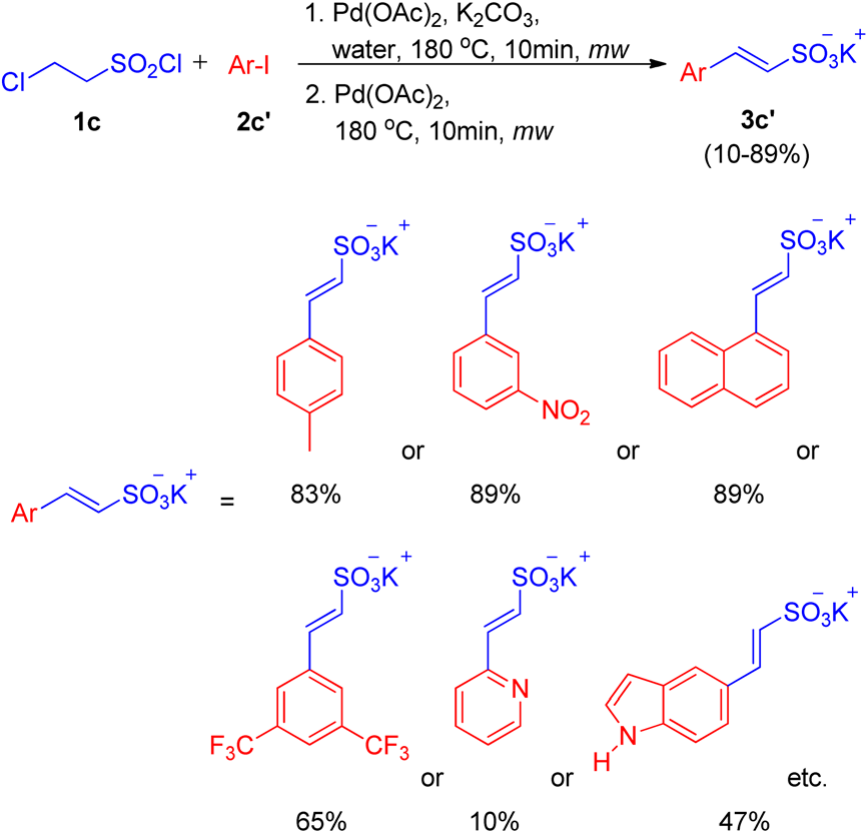
Substrate scope for domino (hydrolysis/dehydrohalogenation/Heck) coupling reaction.

With the optimized conditions in hand *i.e.* usage of 2 mol% Pd(OAc)_2_ as catalyst with 3 equiv. of K_2_CO_3_ as base in water at 180 °C for 10 min under microwave, a variety of haloarenes (2c′; 1 equiv.) were analyzed in a reaction with 2-chloroethanesulfonyl chloride (1 equiv.). Apart from iodobenzene which gave 85% of the isolated styrene sulfonate salt, various substituted iodobenzene displayed a good tolerance towards presence of electron withdrawing and electron-donating groups (Scheme 11) giving moderate to excellent yields (25–89%). On the other hand poisoning of Pd catalyst in case of 2-aminoiodobenzene, 2-iodobenzoic acid and *ortho* substituted heterocycles (2-iodopyridine, 2-iodopyrazine, and 2-iodothiophene) lead to zero to inferior yield of the coupled product (3c′). Other heterocycles such as 5-iodoindole and 3-iodopyridine *etc.* were able to drive the reaction to completion with moderate yield of the olefinated product (47–66%).

The mechanism being followed in this reaction is proposed to initiate with the hydrolysis of sulfonyl chloride to chlorosulfonate under basic conditions followed by it’s dehydrohalogenation at higher temperature to yield the required vinyl sulfonate *in situ* which eventually undergoes coupling with haloarene to form the desired Heck-coupled product.

**Scheme 12:**
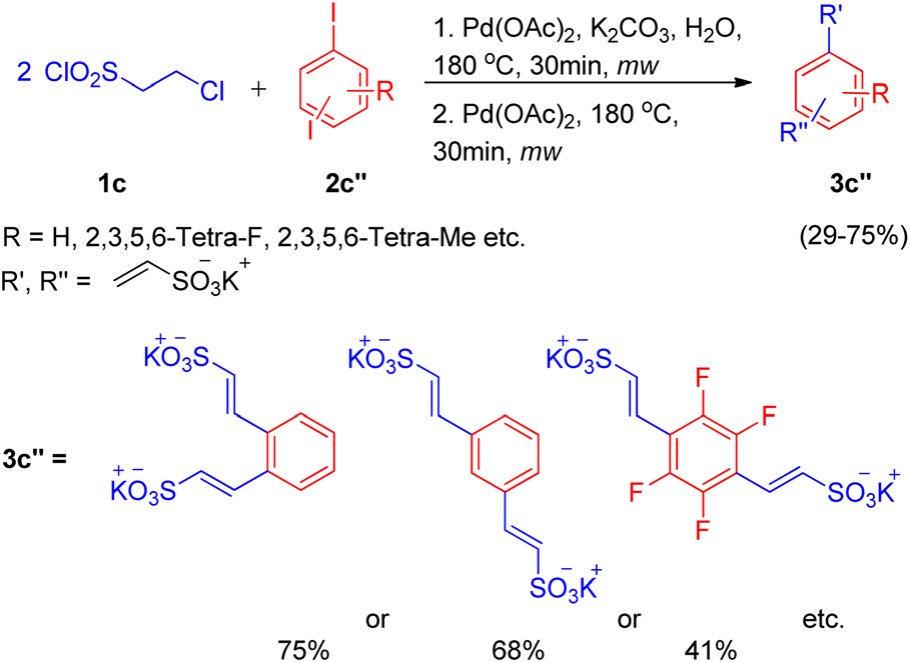
Synthesis of disulfonate salts.

In an attempt to synthesize disulfonate salts (3c′′), the above mentioned protocol was also extended to a number of disubstituted iodobenzenes (Scheme 12; 2c′′), which resulted in generation of disulfonate salts in good yields (29–75%).

**Scheme 13:**
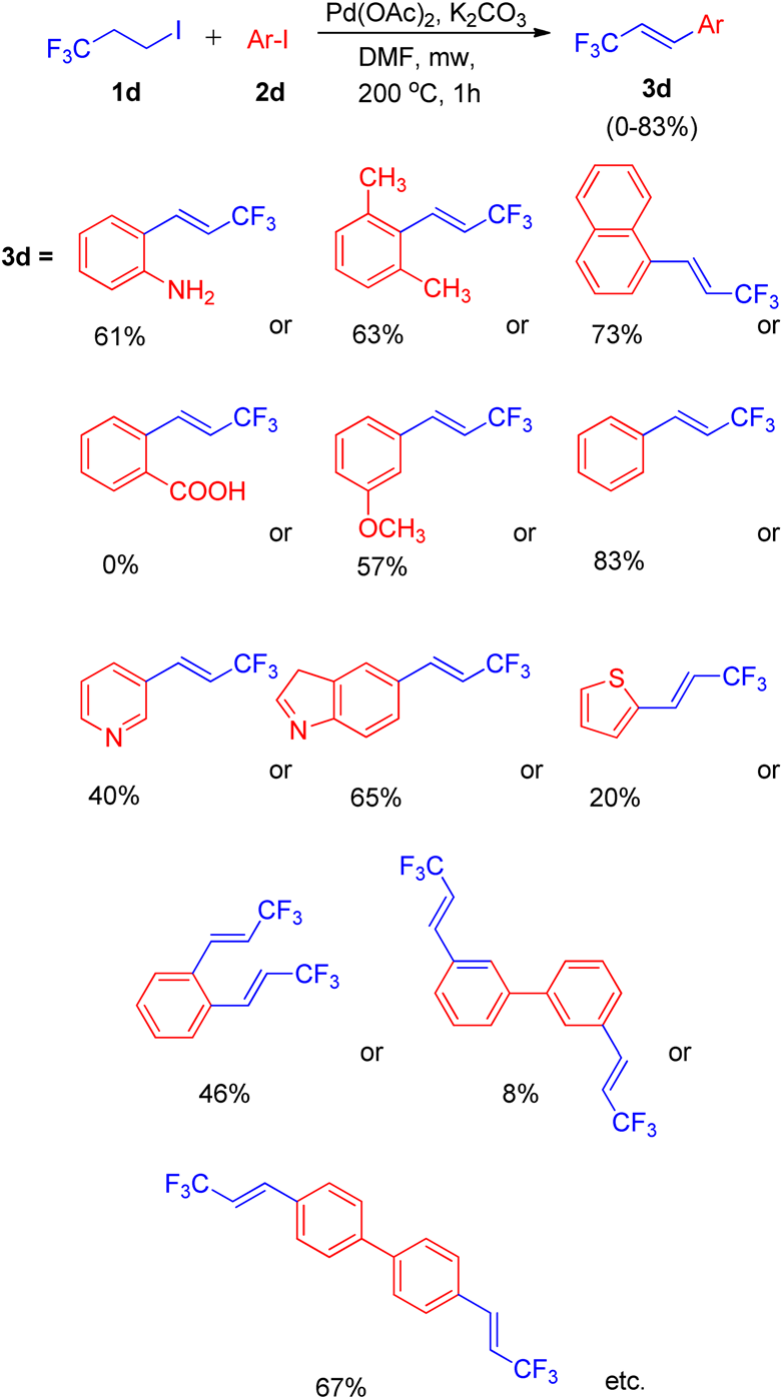
Synthesis of β-trifluoromethylstyrenes, heterocyclic and bis(β-trifluoromethyl)styrenes.

Using a similar domino approach for Heck reaction, G. K. Surya Prakash and co-workers in 2012,^11^ synthesised β-trifluoromethylstyrene derivatives (3d) *via* Heck reaction, in moderate to good yields (52–78%) by using 1-iodo-3,3,3-trifluoropropane (1d) (as an alkene synthon) and iodoarenes (2d) under basic conditions. In order to reach the optimized conditions, numerous screening experiments were performed which revealed that, among various palladium catalysts analyzed, such as Pd(OAc)_2_, Pd_2_(dba)_3_ and Pd(PPh_3_)_2_Cl_2_, and bases such as Li_2_CO_3_, Na_2_CO_3_, K_2_CO_3_, and Cs_2_CO_3_, using Pd(OAc)_2_ (2 mol%) as the catalyst and K_2_CO_3_ (3 equiv.) as base results in maximum yield of the desired product. Thus the reaction between 3-iodotoluene (1 equiv.) and 1-iodo-3,3,3-trifluoropropane (1 equiv.) under optimized conditions *i.e.* using Pd(OAc)_2_ (2 mol%) as catalyst, 3 equiv. K_2_CO_3_ as base in DMF as solvent at 200 °C for 1 h in microwave resulted in formation of desired product with 83% yield (Scheme 13).

Under the above stated optimized conditions, except chlorobenzene, both bromobenzene and iodobenzene were able to form β-trifluoromethylstyrene in 10% and 83% yield respectively. The higher yield of desired product obtained in case of iodobenzene lead to the analysis of various substituted iodobenzenes under optimized reaction conditions. The presence of electron rich substituents in comparison to electron deficient ones on iodobenzene, resulted in a better yield of the desired product and steric effect was found to be ineffective during this protocol (Scheme 13). 2-Aminoiodobenzene, which poisoned the palladium catalyst in the aforementioned protocol (Scheme 11),^10^ was surprisingly able to form the corresponding trifluoromethylstyrene in 52% yield. In addition to substituted iodobenzenes, various heterocyclic iodoarenes as well as diiodoarenes also gave the desired product in low to moderate yield (20–65% and 8–69% respectively).

**Scheme 14:**
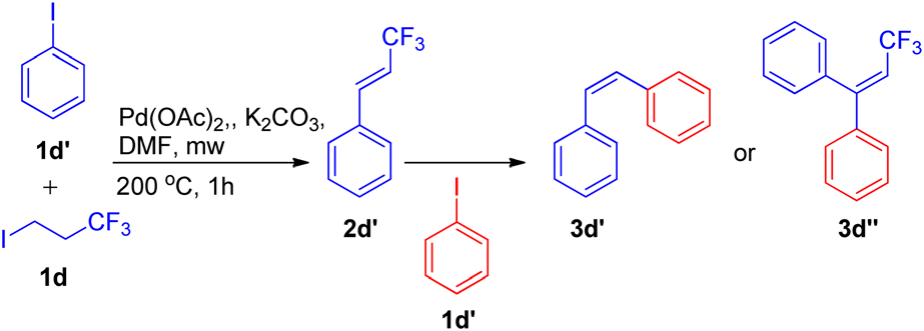
Elimination Heck-domino reaction pathway.

An exclusively-*Z* minor double Heck product (3d′) was also observed during screening of reaction conditions (Scheme 14), however further studies are needed the attain it's optimized conditions.

Thus this method using an elimination/Heck domino reaction sequence offers a simple strategy towards synthesis of β-trifluoromethylstyrene derivatives by avoiding employment of additives and gaseous 3,3,3-trifluoropropene reagent in a phosphine free catalytic system.

Since alcohols are readily available and have received great attention as precursors in various tandem oxidative/dehydrative cross-coupling strategies,^8^ Arun Kumar Sinha and coworkers,^9^ in 2012 reported an approach for the waste-free dehydrative Heck olefination. This approach involved coupling of *in situ* formed styrenes with an aryl halide in ionic liquid. The drawback of secondary alcohols getting converted into carbonyls under Heck-type conditions^11^ and the possibility of cross-contamination while using different medium during dehydration (acidic) of secondary aryl alcohols to convert them into styrene and then using them in Heck (basic) reaction has been overcome by this approach of utilizing ionic liquid where only water is removed as by-product.

**Scheme 15:**
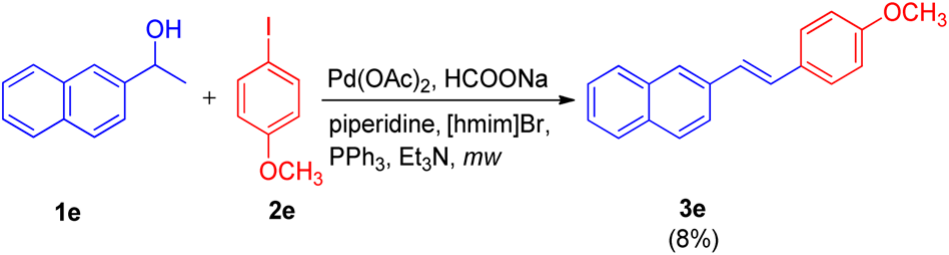
Palladium-catalyzed tendem dehydrative Heck coupling between 4-iodoanisole and 1-(naphthalen-2-yl)ethanol in [hmim]Br ionic liquid.

Initially the reaction of, 4-iodoanisole (2e, 1 equiv.) with 1-(naphthalen-2-yl)ethanol (1e, 1.5 equiv.) in [hmim]Br using Pd(OAc)_2_ (4 mol%) as catalyst, PPh_3_ (5 mol%) as ligand, and Et_3_N (1.5 equiv.) as a base under microwave irradiation (120 W, 150 °C) for 40 minutes was performed (Scheme 15). Analysis of the crude reaction mixture by HPTLC (over HPLC/GC) showed that 3e was obtained in very low quantity (8%). To improve the yield of 3e, a number of organic and inorganic bases such as Bu_3_N, piperidine, DBU, Cs_2_CO_3_, K_2_CO_3_, NaOAc, NH_4_OAc, and HCOONa were rapidly screened through HPTLC, where HCOONa provided 3e in moderate yield (69%). The collaboration of two bases, HCOONa (1.5 equiv.) and piperidine (1 equiv.), in addition to improving the reaction performance, also proved significant in cutting the reaction time in almost half (from 40 minutes to 15 minutes). The usage of an additive LiCl with catalyst [PdCl_2_(PPh_3_)_2_] also increased the yield of 3e to 78%. The crucial role of ionic liquids was established when no reaction was observed in DMF alone.

**Scheme 16:**
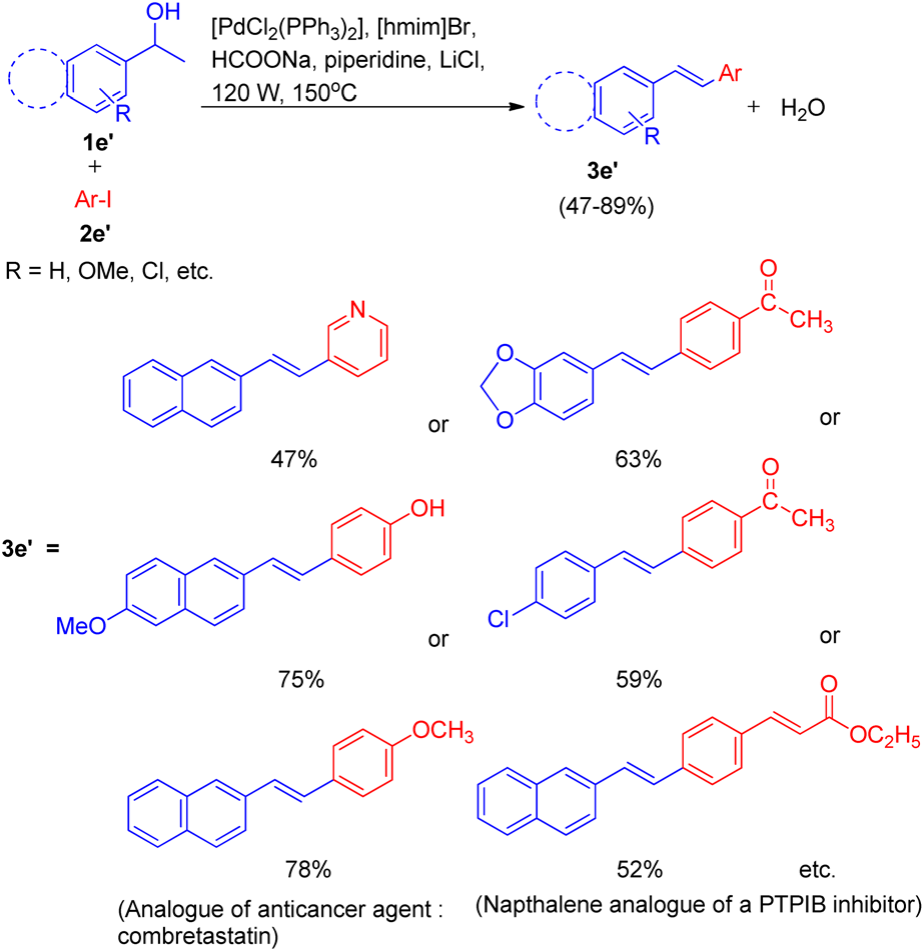
Conversion of secondary aryl alcohols into stilbenoids.

Under the optimized reaction condition the reaction was tried with different secondary alcohols and reaction gave the desired stilbenoids (3e′) in moderate to good yield (47–89%). The products so obtained showed exclusively *E* selectivity (Scheme 16). A naphthalene analogue of a PTPIB inhibitor was also synthesized (52% yield) using this protocol (Scheme 16).

**Scheme 17:**
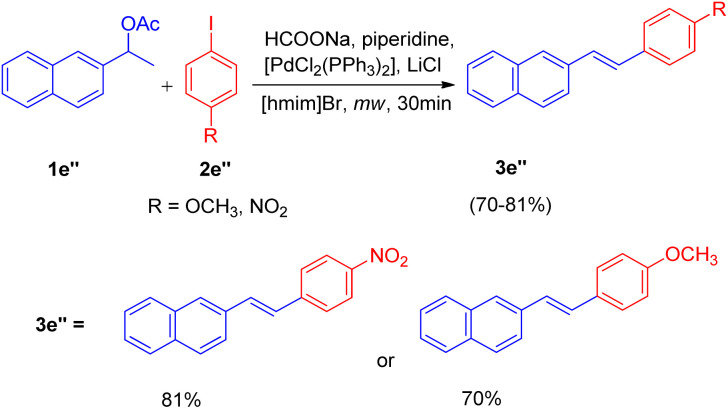
Deacetoxylative Heck coupling.

Motivated by these results, they applied the same strategy for the one-pot deacetoxylative Heck coupling of the acetylated derivative of alcohols (1e′′, Scheme 17) and the corresponding stilbenes (3e′′) were obtained in good yields (70–81%).

**Fig. 3:**
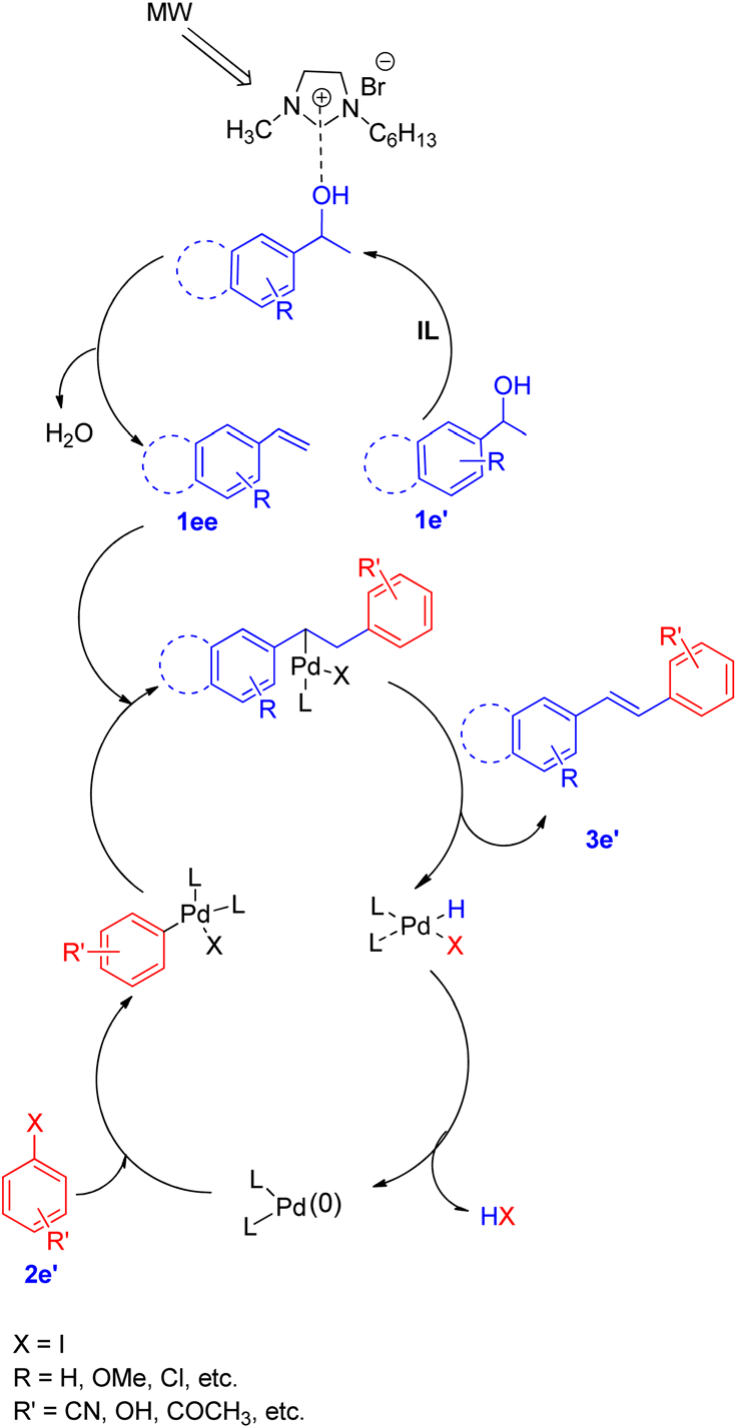
Proposed mechanism for tandem dehydrative Heck coupling in an ionic liquid under microwave irradiation.

A diverse range of stilbene possessing electron withdrawing as well as electron donating groups on either of the 2 substrates (secondary aryl alcohol and aryl halide) can be synthesized using the above stated protocol. This approach provided good yields of the desired stillbene when using polyaromatic alcohols, however, due to the polymerization tendency of the intermediate styrene,^8^ the electron deficient alcohols could only provide required substituted olefins in moderate yield. The proposed mechanism for this protocol has been shown in Fig. 3. The mechanism is proposed to initiate by the microwave and ionic liquid assisted dehydration reaction of 1e′ which leads to formation of styrene (1ee), this styrene then undergoes Pd-catalyzed Heck coupling with aryl halide (2e′) to give the substituted stilbene (3e′). One of the major application of this protocol is it's ability to synthesize numerous analogues of anticancer agent combretastatin^12^ (3e′, 78%) without the requirement of any protection/deprotection step (Scheme 16).

**Scheme 18:**
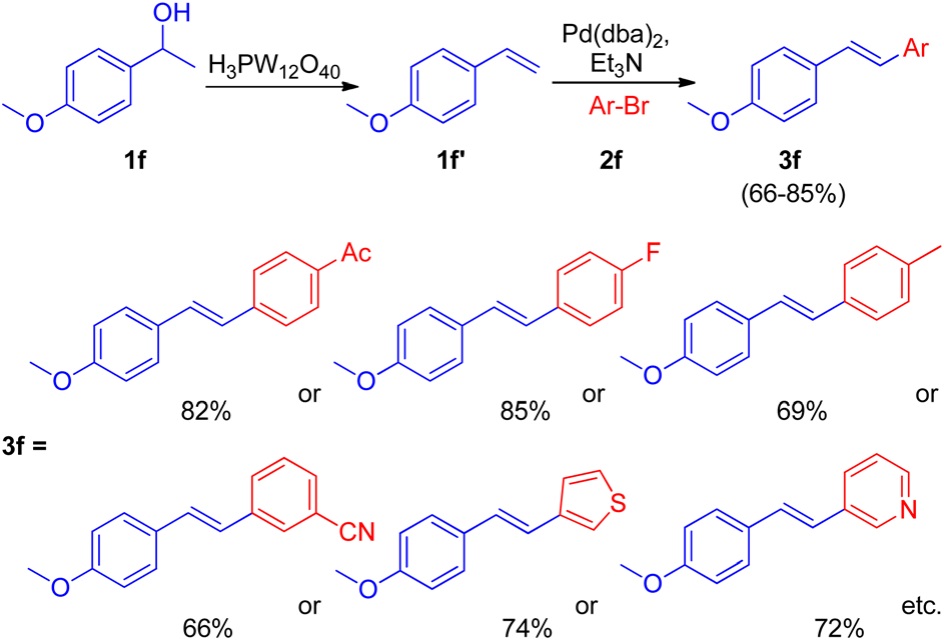
Dehydrative-Heck arylation of benzylic alcohol (1f) with aryl bromides (2f).

After the development of dehydrative Heck reaction in an ionic liquid (acting as solvent) by A. K. Sinha and co-workers,^9^ a similar method was reported by Jianliang Xiao *et al.* in the same year (2012)^13^ with the difference of involving usage of heteropolyacids (HPAs) instead of ionic liquid. The path to achieving optimized conditions began with thermal activation of 1-(4-methoxyphenyl)-ethanol (1f; 1.5 equiv.) in the presence of H_3_PW_12_O_40_ (HPA) in a number of solvents such as DMF, DMA, DMSO, MeCN, hexane, ethanol, anisole, and diglyme *etc.* among which only DMSO was able to drive the dehydration to completion with 55% isolated yield of the desired alkene (1f′). The development of dehydration conditions was followed by screening of reaction parameters for further coupling with aryl halide (2f; 1 equiv.). The neutralization of reaction media caused by the presence of both HPA and base at the same time was overcome by forming a two-step procedure involving dehydration of aryl alcohol (1.5 equiv.) in the presence of HPA in DMSO at 100 °C for 1 h with the subsequent coupling of the alkene formed with aryl halide (1 equiv.) on addition of a base Et_3_N (1.5 equiv.), catalyst Pd(dba)_2_ (2 mol%), and P(*t*-Bu)_3_·HBF_4_ (0.06 equiv.) in DMF at 100 °C for 4 h, resulting in 82% yield of the desired substituted styrene. Various aryl bromides were examined in reaction with benzyl alcohol (1f) under the optimized reaction condition (Scheme 18) and it was found that the aryl bromides containing electron-withdrawing, electron-donating groups as well as heterocyclic substrates were able to form the desired product in good-yields (66–85%).

**Scheme 19:**
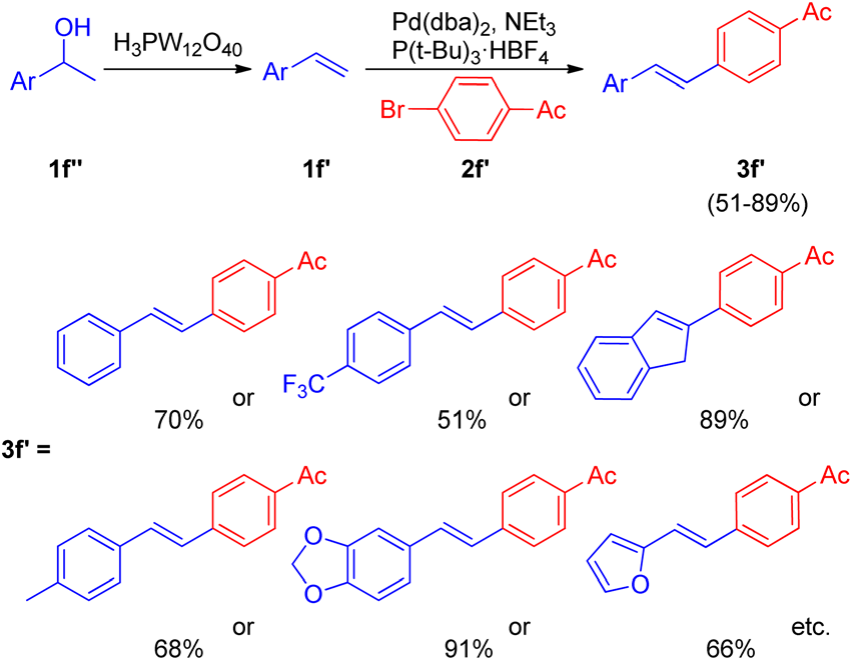
Dehydrative-Heck arylation of alcohols 1f′′ with 4-bromoacetophenone (2f′).

Along with them different secondary aryl alcohols (1f′′) were also analyzed (Scheme 19) by coupling them with 4-bromoacetophenone (2f′), which showed that only electron-rich aryl alcohols were undergoing dehydration under the optimized reaction conditions however a switch from DMSO to diglyme and to 1,2-dichloroethane while using electron-neutral and electron deficient aryl alcohols respectively was necessary to afford good to excellent yield (51–91%) of the corresponding stilbenes (3f′).

Thus the limited substrate scope of secondary aryl alcohol while using ionic liquid as a solvent was overcome to a certain extent through this protocol.

**Scheme 20:**
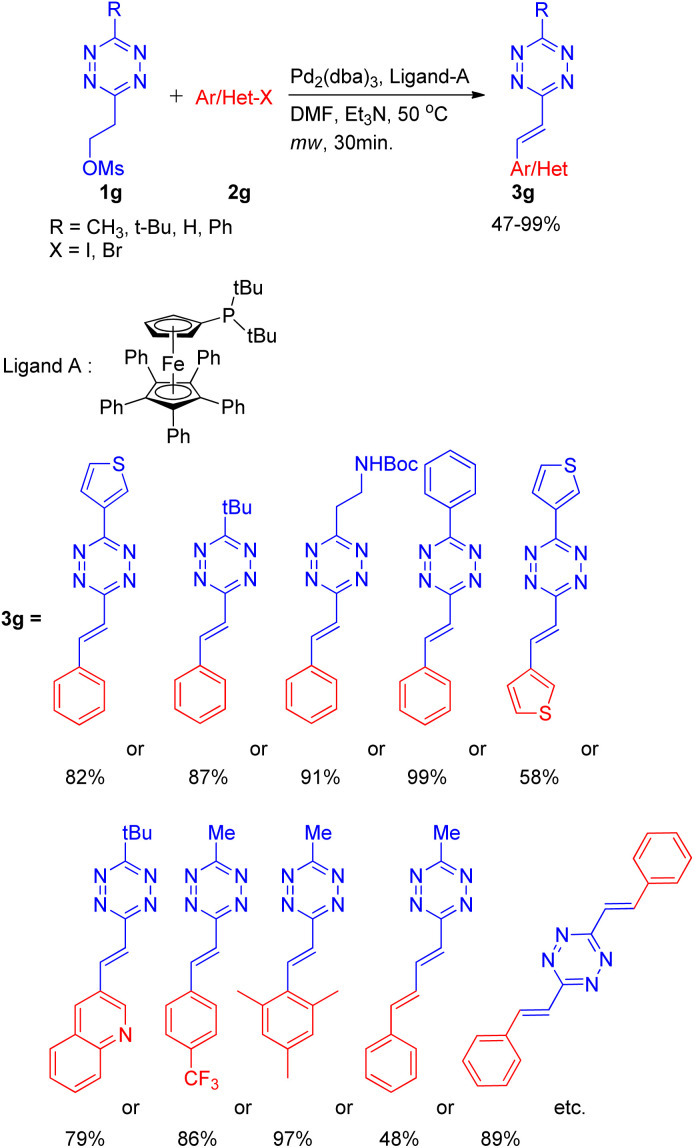
*In situ* synthesis of alkenyl tetrazines for elimination-Heck cascade reaction.

A practical method for the synthesis of 1,2,4,5-tetrazine derivatives, which plays a significant role in live cell and *in vivo* imaging, was developed by K. Devaraj and co-workers^14^ in 2014 by using an elimination-Heck cascade reaction. The screening reaction involved utilization of precursor tetrazine 1g (synthesized by mesylation of 3-methyl-6-hydroxyethyl-1,2,4,5-tetrazine) in place of 3-methyl-6-vinyl tetrazine and reacting it with aryl halide (2g) for an *in situ* elimination-Heck reaction. Screening reactions revealed that among catalysts (Pd_2_(dba)_3_ and Pd(PPh_3_)_4_), and among bases such as NEt_3_ and Cy_2_NMe, using Pd_2_(dba)_3_ (3 mol%), and Cy_2_NMe (3 equiv.) for reaction between precursor 1,2,4,5-tetrazine (1g; 1 equiv.) and halobenzene (2g; 1–1.5 equiv.) along with ligand A (0.12–0.4 equiv.) at 50 °C for 30 min under microwave resulted in maximum yield of the desired product (3g; R = CH_3_; 99%) (Scheme 20).

Under the obtained optimized conditions, the tetrazines with *tert*-butyl, phenyl, heterocycles, and protected amine group *etc.* at position-3 were able to drive the reaction to completion (Scheme 20) giving 3-substituted alkenyl tetrazines in good to excellent yield (82–99%). Similarly, various aryl bromides containing electron withdrawing, electron donating, sterically bulky groups, as well as heterocyclic substituents (Scheme 20) also gave desired product in good to excellent yield (58–97%). Conjugated mono-phenylbutadiene, biphenylbutadiene and bistyryl substituted *s*-tetrazines *etc.* were also synthesized in moderate yield (48–89%) using the same method.

The overall protocol thus generated facilitates the study of highly conjugated 1,2,4,5-tetrazines with the aim of exploiting them in material science, photovoltaics, chemical biology and specifically in live-cell imaging.

In addition to the all the above methods of *in situ* generation of alkenes providing the required Heck coupling product in good to excellent yields, the tandem Wittig–Heck sequence because of the greater availability of aromatic aldehyde and ketones as compared to styrene^15^ and simplicity and efficiency of Wittig reaction, has also been widely researched in the last two decade. Thus the required olefin can either be generated through various procedures given above or by utilizing sequential Wittig–Heck reactions as covered below.

## 3. Mizoroki–Heck reaction between *in situ* generated Wittig alkenes and aryl halides

**Scheme 21:**
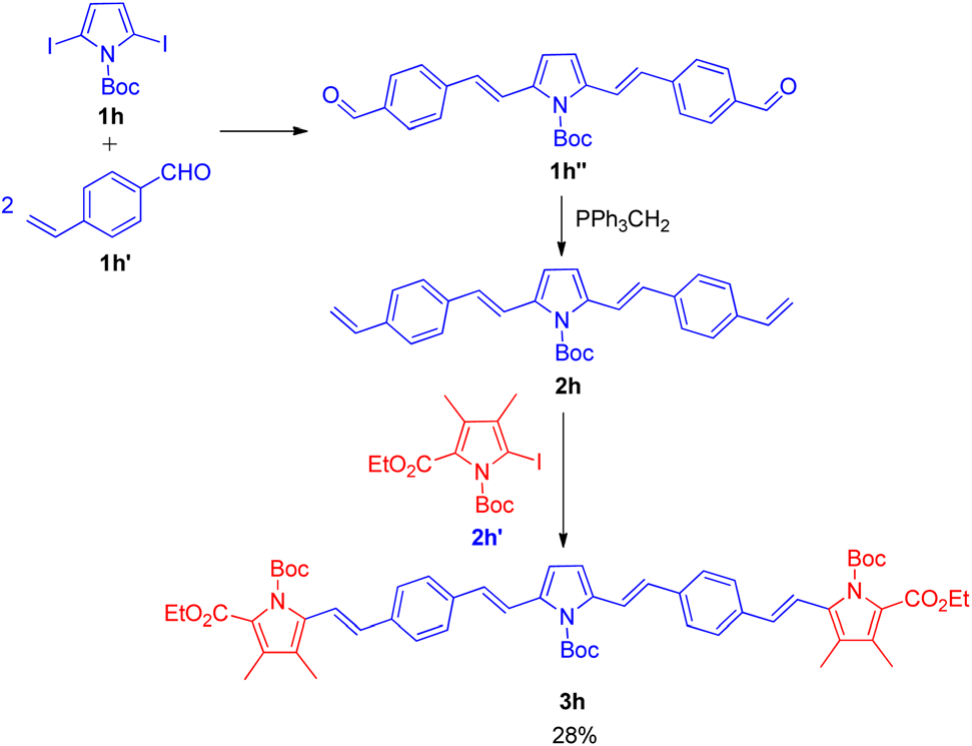
Synthesis of pentacyclic oligomer by a two fold Heck reaction followed by a Wittig reaction and a two fold Heck reaction.

The utilization of sequential Wittig–Heck reaction for formation of substituted olefines began in 2001 when Lutz F. Tietze and co-workers^16^ reported a 2 two-fold Heck reaction *via* one Wittig reaction as one of the method for the synthesis of biologically important linear π-conjugated oligomeric pyrrole derivatives (Scheme 21). The efficient synthesis of various oligomeric pyrrole derivatives connected by divinyl units initiates with two-fold Heck reaction of 2,5-dibromopyrrole (1h; 1 equiv.) derivative with *p*-vinylbenzaldehyde (1h′; 2 equiv.). The pyrrole derivative thus formed (1h′′) undergoes 2-fold Wittig reaction in presence of *n*BuLi (1.1 mol equiv.), and triphenylphosphonium iodide (1.2 mol equiv.) in THF to yield a substituted alkene (2h) which eventually reacts with the iodopyrrole (2h′; 1 equiv.) again in a 2-fold Heck reaction in the presence of 5 mol% Pd(OAc)_2_, potassium acetate (4.0 equiv.), and tetrapropylammonium bromide (1.0 equiv.) in DMF at 75 °C for 2 h under nitrogen or argon atmosphere. They were able to synthesize the desired red pentacyclic oligomer (3h) with 28% yield (Scheme 21). Thus this protocol provided a synthetic pathway to linear π-conjugated oligomeric pyrrole derivatives with up to 5 arenes, however the method can also be exploited for the formation of higher oligomers.

Owing to the presence of medium sized heterocyclic rings fused to aryl rings in many natural products,^17^ K. C. Majumdar and co-workers^17^ in 2008, developed an efficient system with high regioselectivity and high yield for the synthesis of oxepin ring (3i), having significance in the preparation of naphthoxepin derivatives (antipsychotic drugs^17^), by employing sequential Wittig and intramolecular Heck reaction. For the intramolecular Heck reaction to take place, the required substrate (2i) was generated *via* Wittig reaction of substrate 1ii generated through reaction between hydroxy-aldehydes(1i′) (synthesized *via* Reimer–Tiemann reaction of 1i), with either 2-bromobenzyl bromide(1i′′) or 2-bromo-5-methoxy benzyl bromide(1i′′′). This reaction proceeded in dry acetone with anhydrous potassium carbonate as base and a small amount of sodium iodide.

**Scheme 22:**
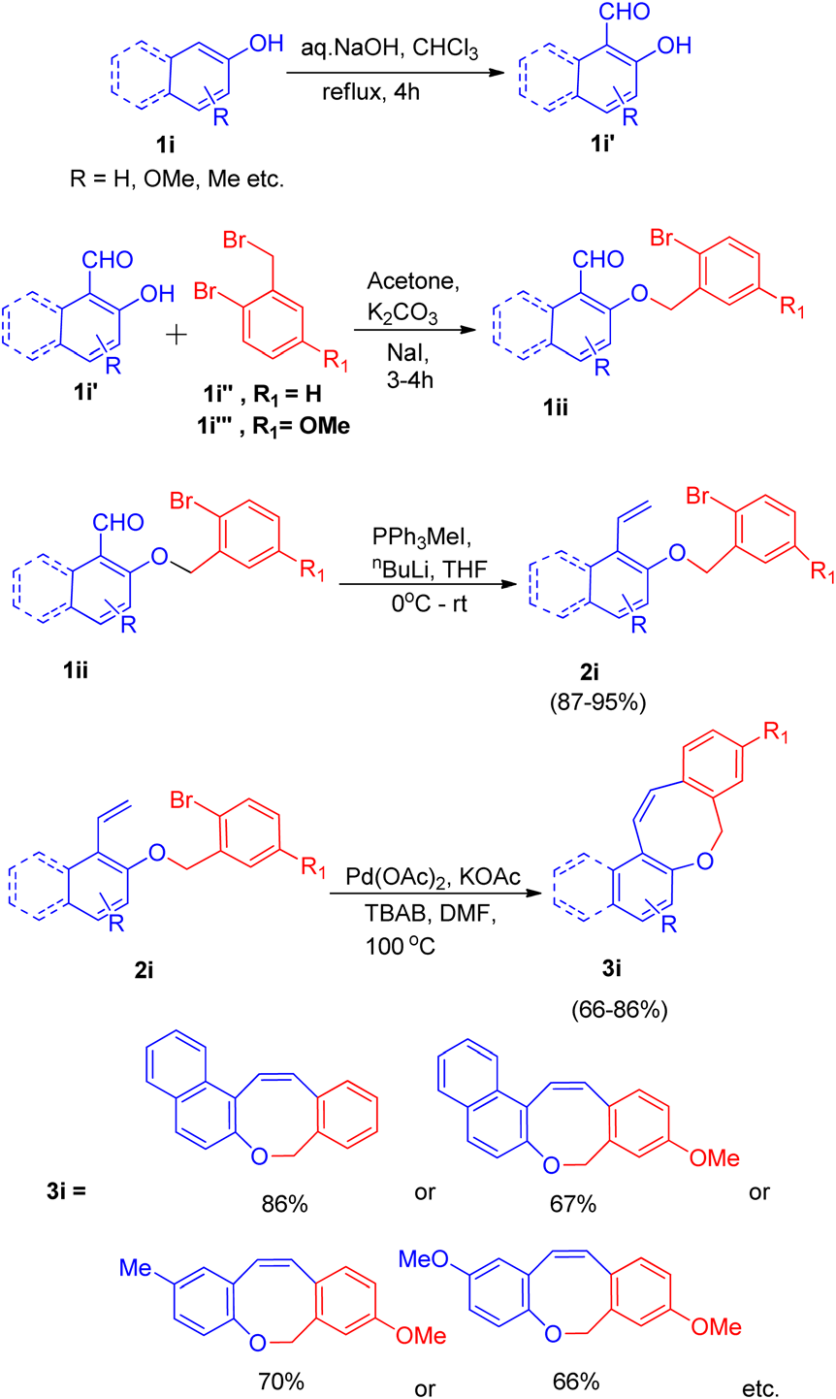
Palladium-catalyzed intramolecular Heck cyclization of 2i (synthesized *via* Wittig reaction of 1ii) to form 3i.

The product (1ii) through Wittig reaction generates 2i, which then results in formation of the *Z*-isomer of eight-membered naphthoxocine compound (3i) with excellent yield (upto 86%) under Heck reaction condition (Scheme 22). Various screening experiments were performed to get optimized reaction condition for Heck reaction revealed that among Pd(PPh_3_)_2_Cl_2_, Pd(OAc)_2,_ PdCl_2_ and Pd(PPh_3_)_4,_ Pd(OAc)_2_ gave the desired product in excellent yield. The presence of TBAB also proved to be necessary for the reaction to go to completion since in the absence of it, no coupling product was obtained.

Among various bases which were analyzed such as K_2_CO_3_, Et_3_N, Ag_2_CO_3_, KOAc, Cs_2_CO_3_, and solvents such as DMF, CH_3_CN, Et_3_N, dioxane, KOAc as base and DMF as solvent was found to be the most efficient ones. Thus for the intramolecular Heck reaction in 2i, on providing a nitrogen atmosphere containing Pd(OAc)_2_ acting as a catalyst, KOAc as a base, and TBAB as an additive in dry DMF as solvent for 2 h at 100 °C (Scheme 22) the desired eight-membered cyclized oxocine derivatives were obtained in moderate to good yield (66–86%). Similarly *Z*-isomers of other 8-membered oxocine derivatives were also prepared *via* 8-endo trig cyclization. The efficiency of these sequential Wittig–Heck reactions were increased by reducing the reaction time and work-up steps.^12^ Fused oxocine derivatives (3i) can be formed in high yield through this simple cyclization protocol.

**Scheme 23:**
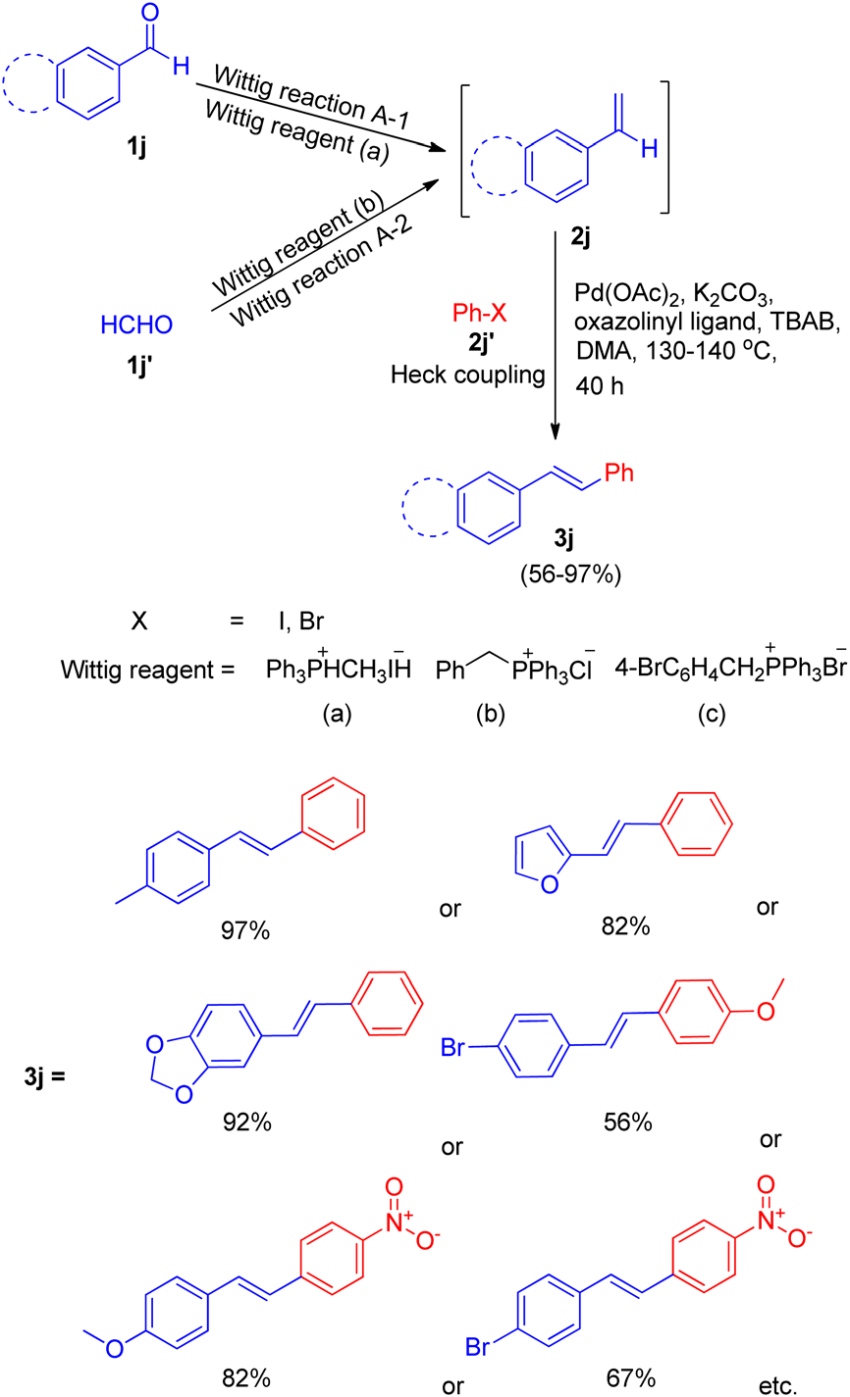
Proposed routes of *in situ* synthesis of styrene through Wittig reaction for subsequent one-pot Heck reaction.

One among the tactic for this sequential-Wittig process was also given by Akeel S. Saiyed and Ashutosh V. Bedekar in 2010 *via* one pot process.^5^ The main feature of this method is the *in situ* synthesis of required olefin. Olefins (2j) were synthesized by Wittig reaction from aldehyde (1j, 1j′) and acceptable phosphonium salt (Scheme 23). The two ways (A-1, A-2) shown in Scheme 23 for *in situ* synthesis of styrene ends with formation of stilbene on providing Mizoroki–Heck conditions. In this approach, aromatic aldehyde (1j) with phosphonium salt undergo Wittig reaction even with weak base like potassium carbonate, resulting in formation of an olefin (2j) to further couple with an aryl halide (2j′) under Heck reaction conditions (Scheme 23).

**Fig. 4:**
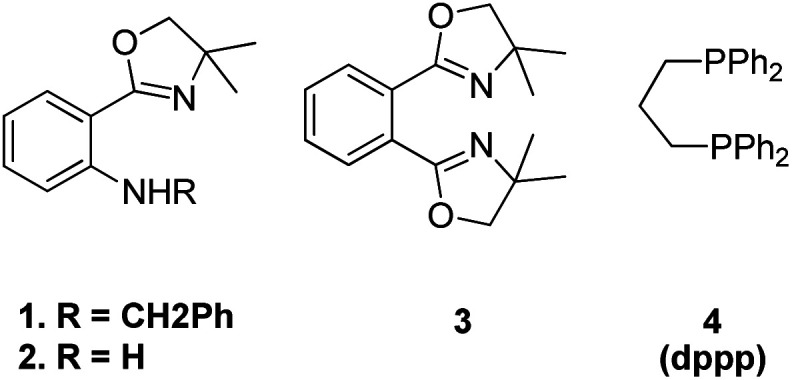
Ligands investigated for the one-pot approach for Wittig–Heck reaction.

The optimized conditions for this protocol include employing 1 equiv. of aldehyde with 1 equiv. of phosphonium salt for wittig reaction. The desired alkene thus formed then undergoes Heck coupling with 1 equiv. of an aryl halide in the presence of catalytic quantity of Pd(OAc)_2_ (0.5 mol%), excess of K_2_CO_3_ (3.5 equiv.), 0.01 equiv. of oxazolinyl ligand (1 or 2) (Fig. 4), and TBAB (0.2 equiv.) (as phase transfer catalyst) which was heated in DMA at 130–140 °C for 40 h to result in formation of desired *trans*-stilbene (3j) in good yields (56–94%). The presence of electron donating or electron withdrawing groups on either of the substrate does not seem to affect the reaction yield to a significant extent making this approach for *in situ* olefination an attractive option. However when the reaction was carried out using paraformaldehyde and 4-BrC_6_H_4_CH_2_PPh_3_Br as phosphonium salt, with either 4-iodoanisole or 1-bromo-4-nitrobenzene, the yield of the substituted olefin decreased to 56 and 67% respectively.

**Scheme 24:**
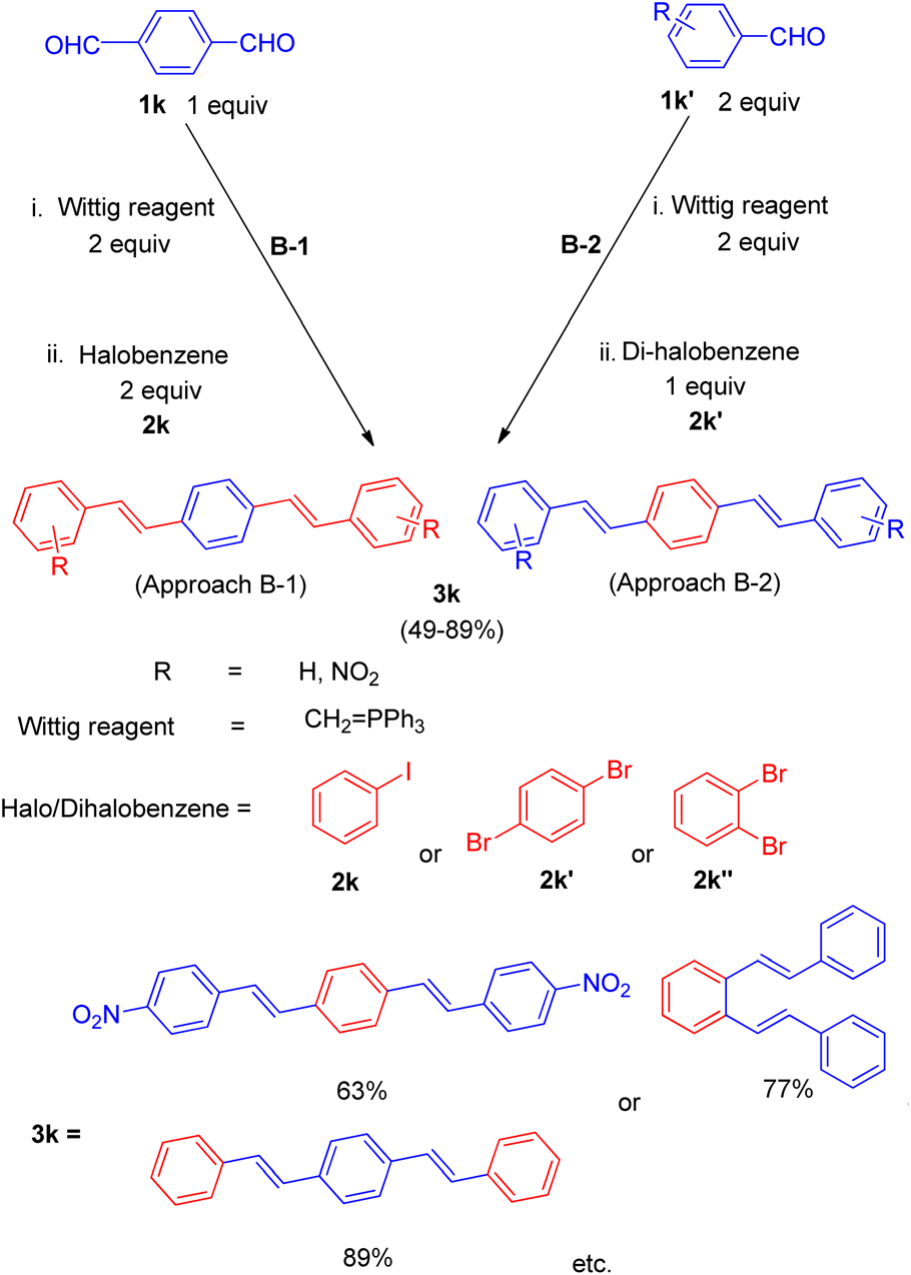
One-pot five-component approach for the synthesis of distyryl benzene derivatives through Wittig–Heck reaction.

They also gave a one pot five component approach involving simultaneous formation of two double bonds between three aromatic rings through a combination of Wittig and Mizoroki–Heck reaction in a single step process (Scheme 24).

This one-pot five component process was done in two ways under similar reaction conditions: the 1st method involves *in situ* preparation of 1,4-divinyl benzene by the reaction of 1 equiv. of terephthalaldehyde (1k) and 2 equiv. of Wittig reagent and subsequent introduction of this into the palladium catalyzed Heck reaction for coupling with 2 equiv. of halobenzene (2k) (approach B-1, Scheme 24) while the 2nd method of making 1,4-divinyl benzene involves making two fold excess of styrene by using 2 equiv. of benzaldehyde (1k′) with 2 equiv. of Wittig reagent and subsequent introduction of this into the palladium-catalyzed Heck reaction for coupling with 1,4-dibromo benzene (2k′) (approach B-2, Scheme 24). For 2nd approach, 1,2-dibromobenzene (2k′′) can also be exploited to yield the desired distyryl benzene with 77% yield. The overall yield of the reactions conducted in a single pot was very good, either with ligand 1 or with ligand 2 and with 1,3-bis(diphenylphosphino)propane(dppp) ligand 4 (Fig. 4). Other distyryl benzenes using different aryl halides, and aromatic aldehydes, were also prepared by exploiting the same approach in good to excellent yield (49–89%).

The one-pot methods described here have the advantages of requiring fewer work-up and purification steps, providing appropriate chemical yield, and minimizing waste caused by polymerization of intermediates.

**Scheme 25:**
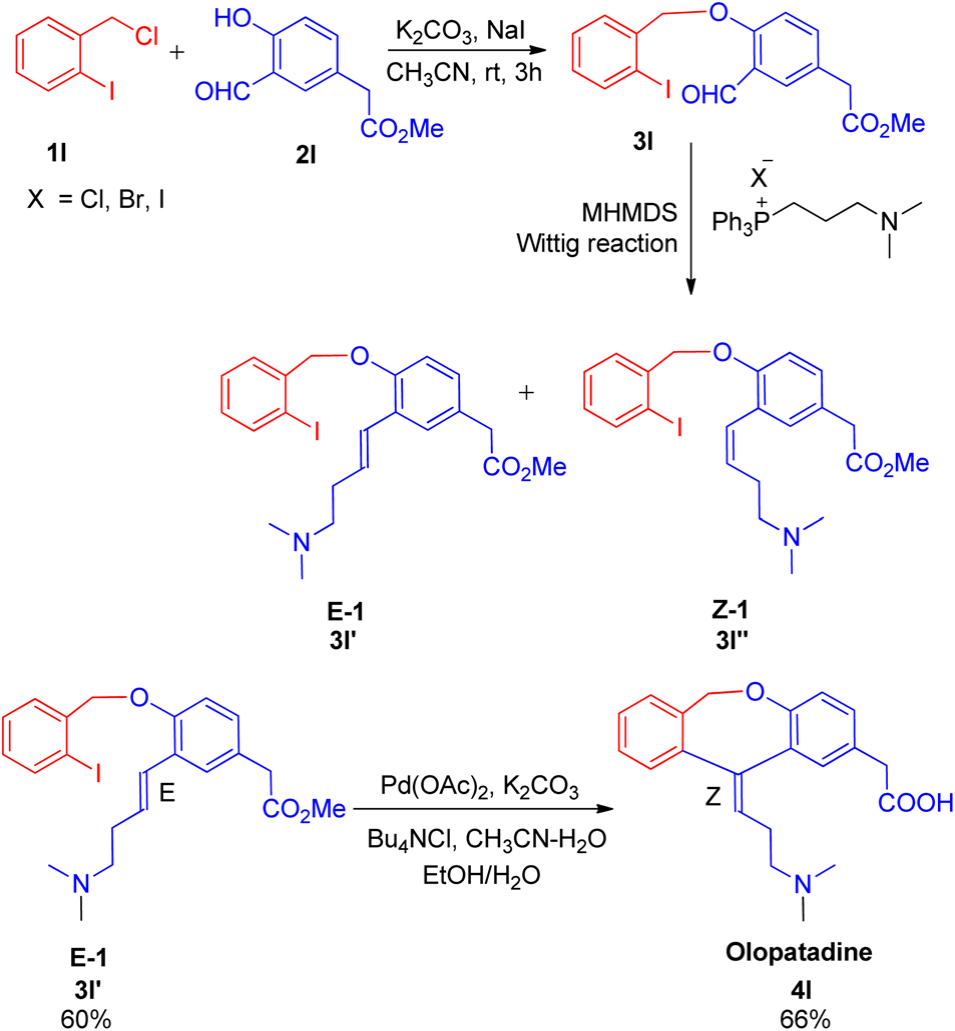
Williamson reaction between 1l and 2l followed by sequential Wittig–Heck reaction of 3l for the synthesis of antihistaminic drug olopatadine (4l).

Joan Bosch *et al.* in 2012,^18^ developed a synthetic path for the synthesis of olopatadine, an antihistaminic drug and it's *E*-isomer having sequential highly stereoselective Wittig–Heck reaction as a key step in the process. The protocol for drug development initiates from Williamson reaction to assemble the benzyl aryl ether moiety (3l), which then undergoes Wittig olefination followed by successive intramolecular Heck coupling. The required aldehyde for Wittig reaction was 1st formed by Williamson reaction between 1l and 2l by adding a solution of 2l (1 equiv.) in acetonitrile to a mixture of substituted iodobenzene (1l; 1 equiv.), K_2_CO_3_ (1.1 equiv.), and NaI (0.25 equiv.) in acetonitrile, at room temperature for 3 h. The corresponding aldehyde (3l) thus generated was then treated with phosphonium salt to form the olefin (*E*-isomer; 3l′) which ultimately undergoes intramolecular Heck coupling in presence of Pd(OAc)_2_ (20 mol%), Bu_4_NCl (1 equiv.) K_2_CO_3_ (2.52 equiv.) as base, in acetonitrile–water (10 : 1) mixture followed by alkaline hydrolysis, to yield olopatadine drug (4l; Scheme 25) with complete stereoselectivity. Following a similar reaction pathway and by utilizing aldehyde prepared from 2-formylbenzyl bromide and 4-hydroxy-3-iodophenylacetic ester, *trans*-olopatadine (*E*-isomer of 4l) was also synthesized in 70% yield with complete stereoselectivity. On the contrary, the intramolecular Heck reaction using the cis isomer (3l′′) resulted in a mixture of *Z* and *E*-isomers of olopatadine drug. The stereoselectivity of the product generated is highly affected by the selection of base taken during ylide formation from phosphonium halide. Using lithium base LHMDS instead of KHMDS during ylide formation from phosphonium iodide shifted the stereoselectivity of the Wittig reaction from obtaining *E*/*Z* alkenes in a 1 : 3 ratio to 9 : 1 with 73% yield. Thus this methodology involving highly stereoselective sequential Wittig–Heck reaction provided a synthetic route to antihistamine drug (4l) and it's *E*-isomer.

**Scheme 26:**
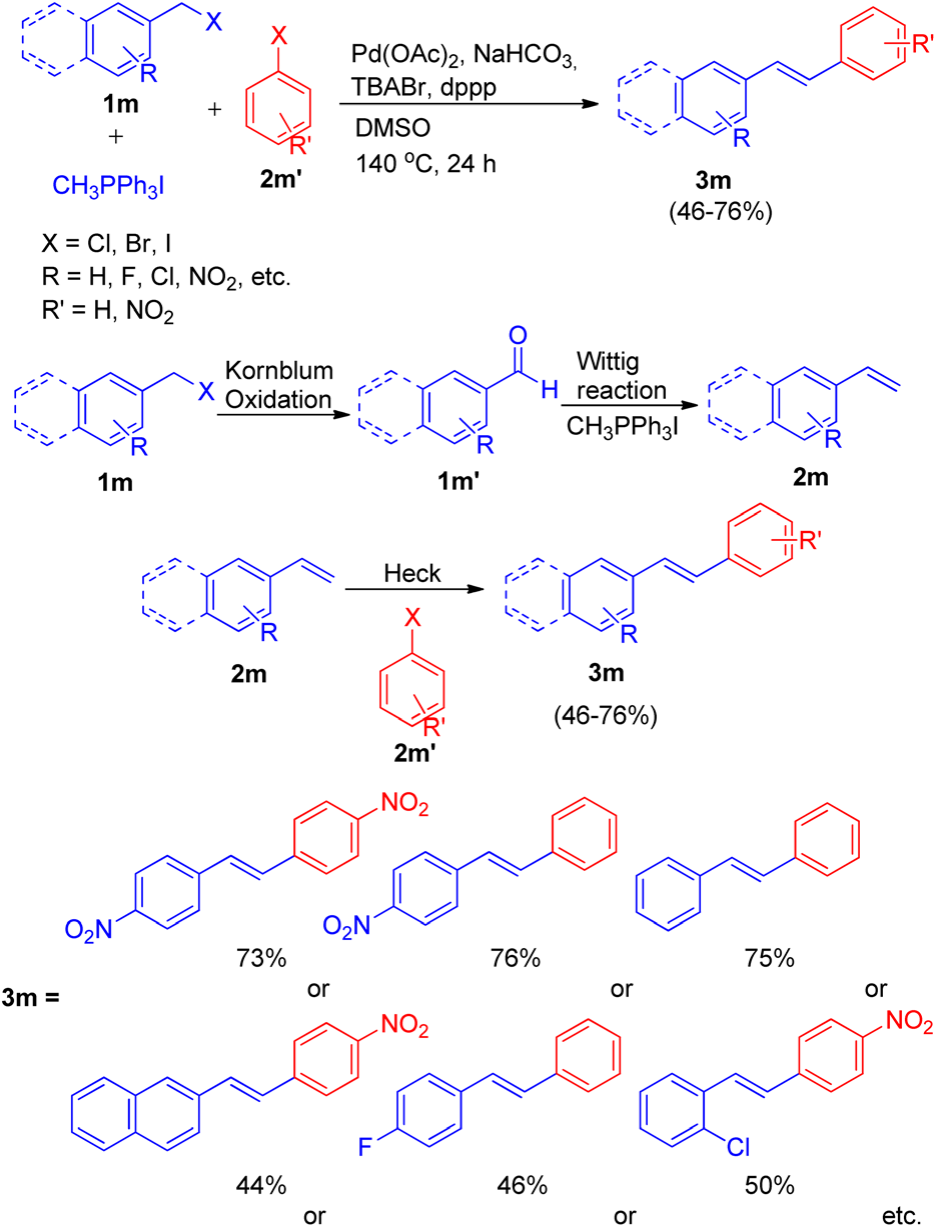
Synthesis of unsymmetrical stilbenes from benzyl halide by one-pot oxidation-Wittig–Heck sequence.

Ashutosh V. Bedekar and co-workers^19^ in 2012 also reported one-pot Wittig–Heck reaction for the formation of stilbenes, by utilizing aromatic aldehyde (1m′) synthesized from Kornblum oxidation of benzyl halide (1m) (Scheme 26). The complete reaction sequence involved oxidation of benzyl halide (1m) to aldehyde (1m′), which then undergoes Wittig reaction to produce corresponding olefin (2m) in presence of a phosphonium salt. Further this olefin undergoes palladium-mediated Mizoroki–Heck reaction with an aryl halide (2m′) to produce *E*-isomers of stilbene (3m) in moderate to good yield (46–76%).

The optimized condition for this protocol involves utilization of 1.5 equiv. of benzyl halide for Kornblum oxidation to result in formation of an aromatic aldehyde. Further reaction of this aromatic aldehyde with 1.5 equiv. of one-carbon Wittig salt along with 1.0 equiv. of aryl halide for sequential Wittig–Heck reaction in the presence of 1 mol% Pd(OAc)_2_ as catalyst, 6.0 equiv. of NaHCO_3_ as base, 0.2 equiv. of TBABr, 2% dppp in DMSO as solvent at 140 °C for 24 h yields the required unsymmetrical stilbenes, with primarily *E*-isomers, in moderate to good yield (46–76%). A variety of benzyl halides and aryl halides bearing EWGs were analyzed under these optimized conditions and it was observed that except for the reaction of 1-(bromomethyl)-4-nitrobenzene with 1-bromo-4-nitrobenzene and with iodobenzene, giving the desired products in 73 and 76% respectively (Scheme 26), the presence of electron withdrawing on either of the two substrate or on both, decreases the yield of the corresponding stilbenes significantly.

**Scheme 27:**
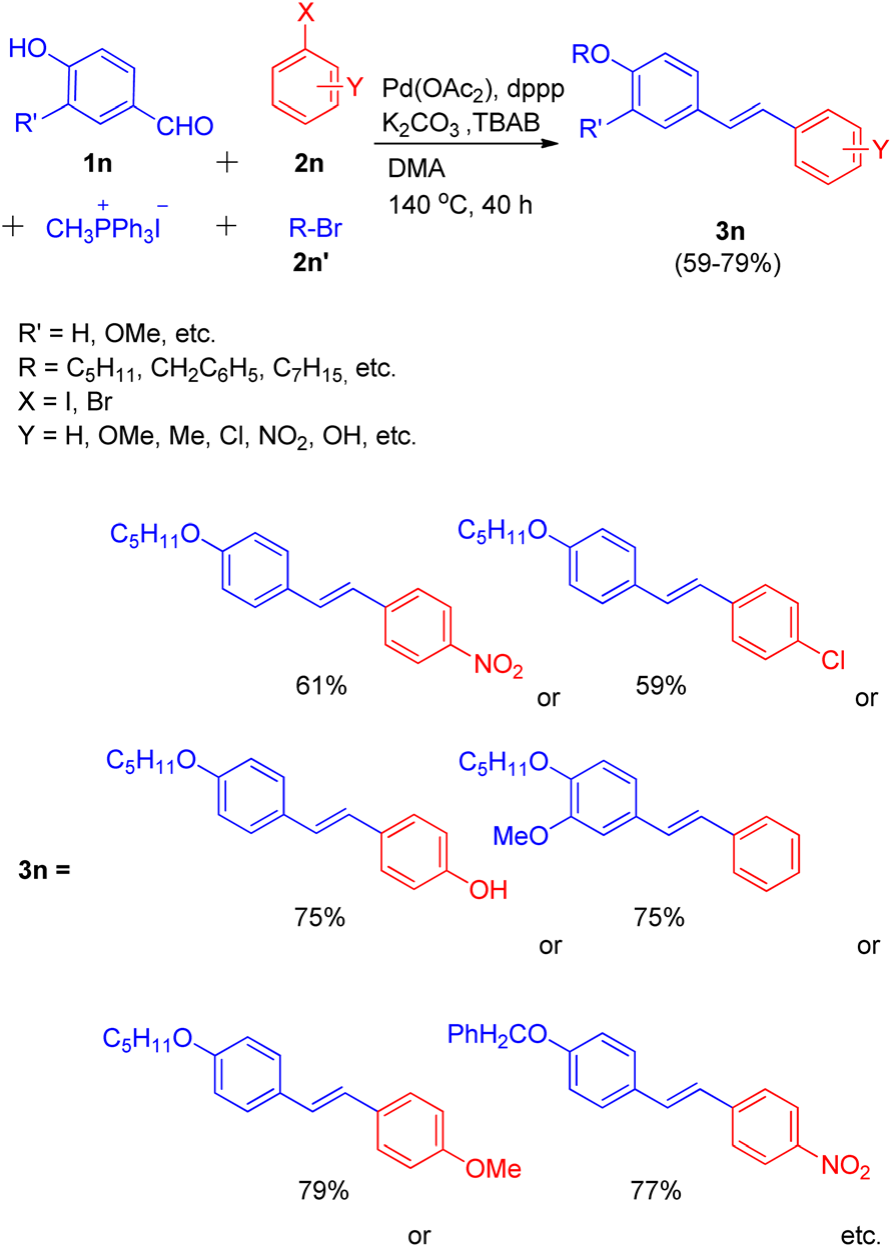
One-pot path for the synthesis of *O*-alkyloxystilbene through *O*-alkylating Wittig–Heck reaction.

In the same year, they were also able to develop a novel one-pot method for the synthesis of highly conjugated alkyloxy stilbenes (3n) from hydroxy benzaldehyde (1n) and one carbon Wittig salt *via* tandem O-alkylating Wittig–Heck reaction.^20^ The method involved palladium-catalyzed Mizoroki–Heck reaction (Scheme 27) of alkyloxystyrene (generated *in situ via* Wittig reaction) with an aryl halide (2n).

The given approach involves synthesis of alkyloxystyrene *via* Wittig reaction of hydroxybenzaldehyde with simultaneous alkylation of the hydroxy group. This is then followed by Heck coupling of the *in situ* generated alkyloxystyrene with readily available functionalized aryl halide to ultimately result in the formation of corresponding stilbene.

Using different aldehydes and alkyl halides, various screening experiments were performed in order to get an optimized reaction condition which revealed that 1.2 equiv of phosphonium salt for Wittig reaction of 1.2 equiv. of aldehyde, along with 1.2 equiv. alkyl halide for alkylation, and 1.0 equiv. of aryl halide for Heck coupling in the presence of Pd(OAc)_2_ (0.5%) as catalyst, 6 equiv of K_2_CO_3_ as base, 1.0% dppp, 10% TBAB in DMA as solvent at 140 °C for 40 h resulted in moderate to good yield (59–79%) of the mostly *E*-isomer of corresponding alkyloxystilbene (Scheme 27).

Various aryl halides were analyzed for the above mentioned protocol and it was found that except for the reaction of 1-bromo-4-nitrobenzene and 1-bromo-4-chlorobenzene with 4-hydroxybenzaldehyde and 1-bromopentane, the presence of electron rich and electron poor groups on aryl halide does not affect the overall yield of the reaction.

**Scheme 28:**
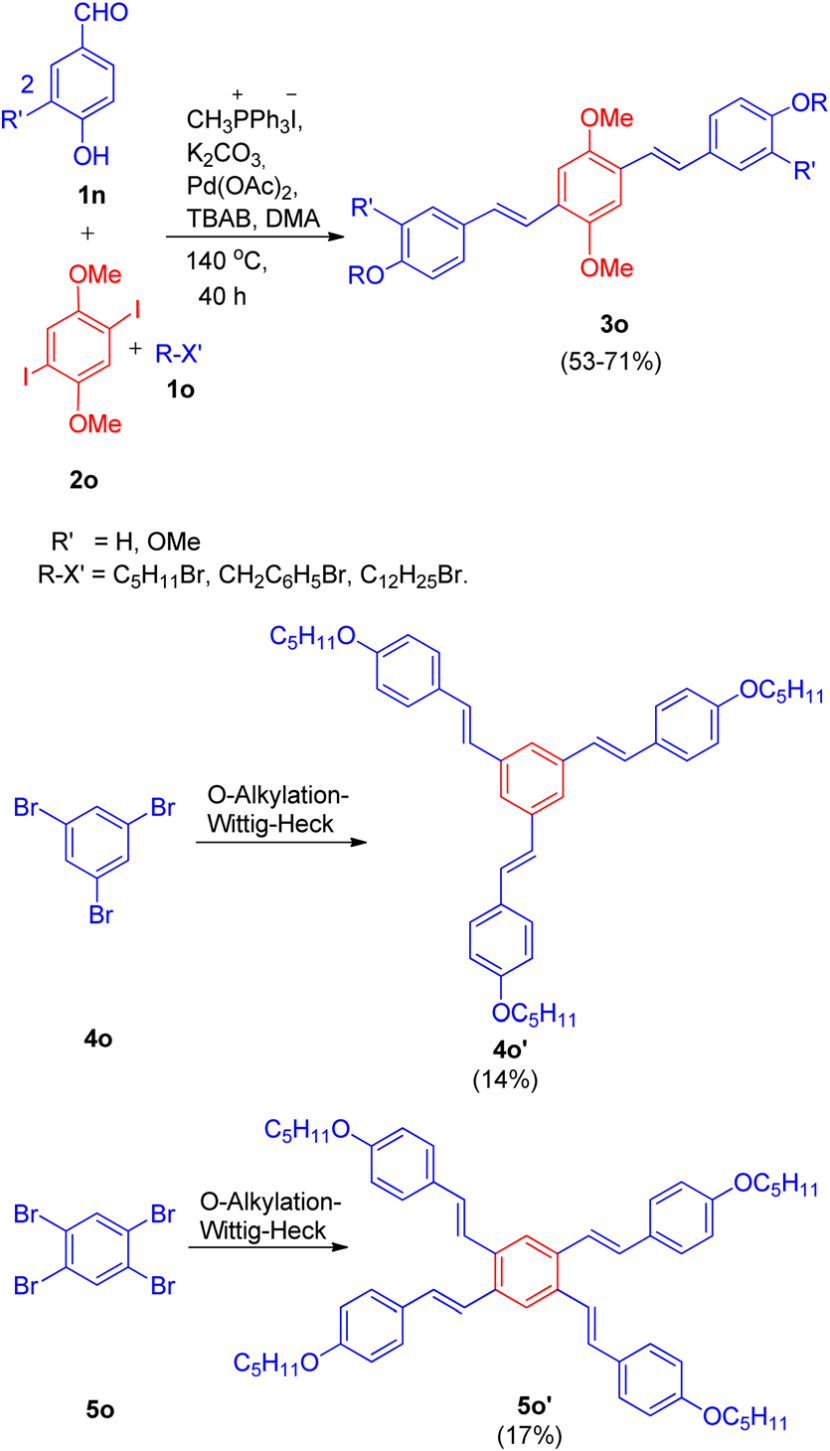
Synthesis of distyrylbenzene, tri- and tetra-stilbene derivatives (3o, 4o′ and 5o′ respectively) through one-pot Wittig–Heck reaction.

This method was also employed for the generation of primarily *E*, *E* isomers of distyrylbenzenes(3o) having C6–C2–C6–C2–C6 framework (Scheme 28) by reacting 2,5-dimethoxy-1, 4-diiodobenzene (2o) with the same hydroxybenzaldehyde (1n) with one-carbon phosphonium salt and appropriate alkyl halide (1o). This one-pot reaction sequence also gave the required product in good yields (53–71%). Similarly tri- and tetra-stilbene derivatives (4o′ and 5o′ respectively) by exploiting 1,3,5-tribromobenzene (4o) and 1,2,4,5-tetrabromobenzene (5o), were also formed (Scheme 28), but with very low yield (14–17%).

**Scheme 29:**
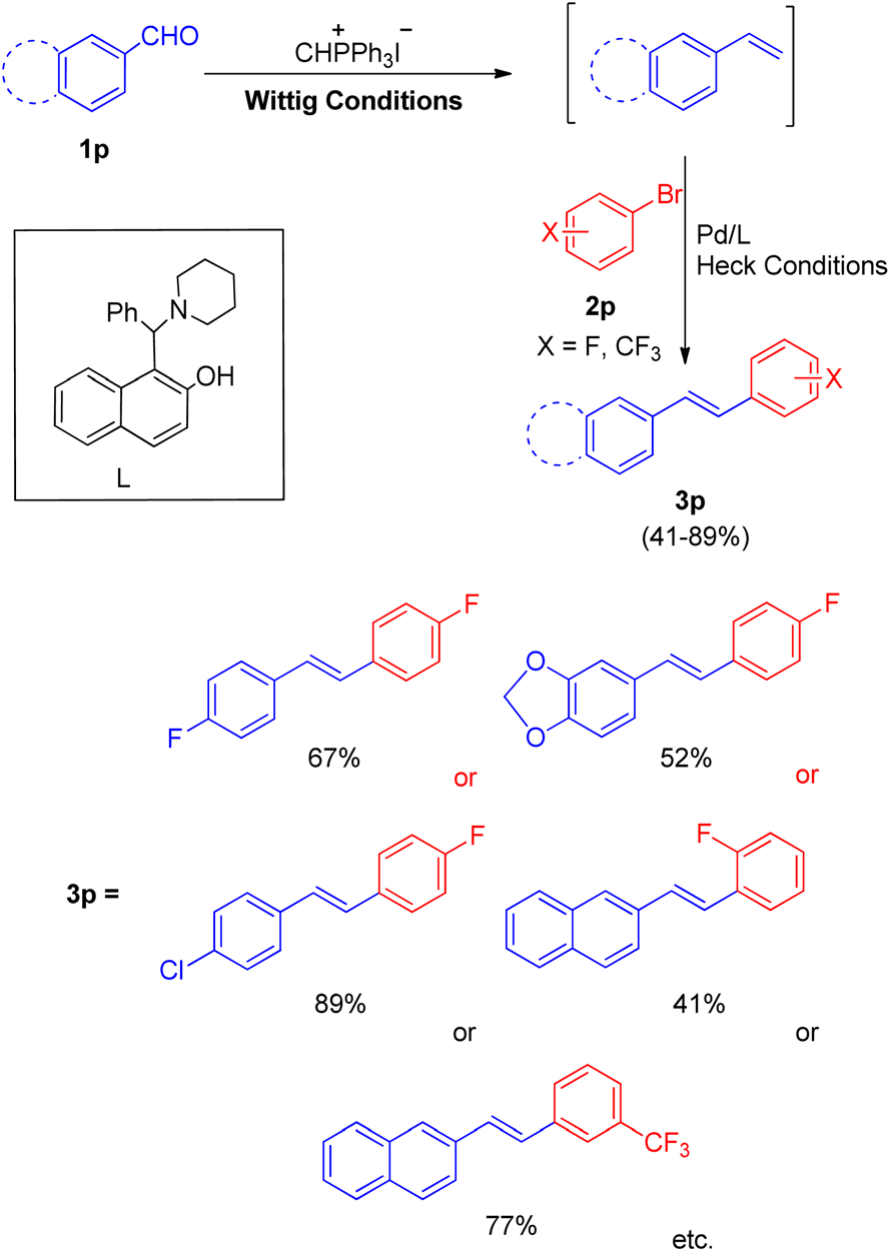
Synthesis of fluorinated stilbenes through Wittig–Heck reaction.

Soon after that in 2012, a phosphine free one-pot Wittig–Heck reaction for the synthesis of varied fluorinated styrylbenzene derivatives^21^ was also developed by them. The reaction between various aromatic aldehyde (1p) with Wittig salt and base provided the required olefin (1p′) (generated *in situ*) for further carrying out the Mizoroki–Heck reaction with an aryl halide (2p) to form variety of fluorinated stilbene derivatives (Scheme 29; 3p). The optimized conditions for this protocol include employing 1.5 equiv. of aromatic aldehyde, 1.5 equiv of CH_3_PPh_3_I for Wittig reaction to yield the desired olefin for further coupling with Ar–X (1.0 equiv.), in the presence of Pd(OAc)_2_ (0.5 mol%) as catalyst, 1-(a-aminobenzyl)-2-naphthol (6 mequiv.) as ligand (L), K_2_CO_3_ (4.0 equiv) as base, with 0.2 equiv. of TBAB, in DMA as a solvent, under N_2_ atm, at 140 °C for 40 h to synthesize corresponding fluorinated styrylbenzene derivatives (3p) with moderate to good yield (41–89%).

These fluorinated stilbene derivatives were then efficiently used for the synthesis of various fluorinated polyaromatic hydrocarbons. The electronic environment of the groups present on either of the substrate did not seem to affect the reaction yield. Thus this path developed by them provides a wide scope of generating fluorinated polyaromatic hydrocarbons (F-PHCs) having significance in cancer research.^17^

**Fig. 5:**
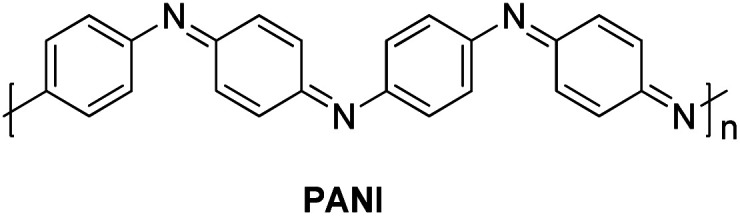
Structure of PANI utilized in one-pot Wittig–Heck reaction.

**Scheme 30:**
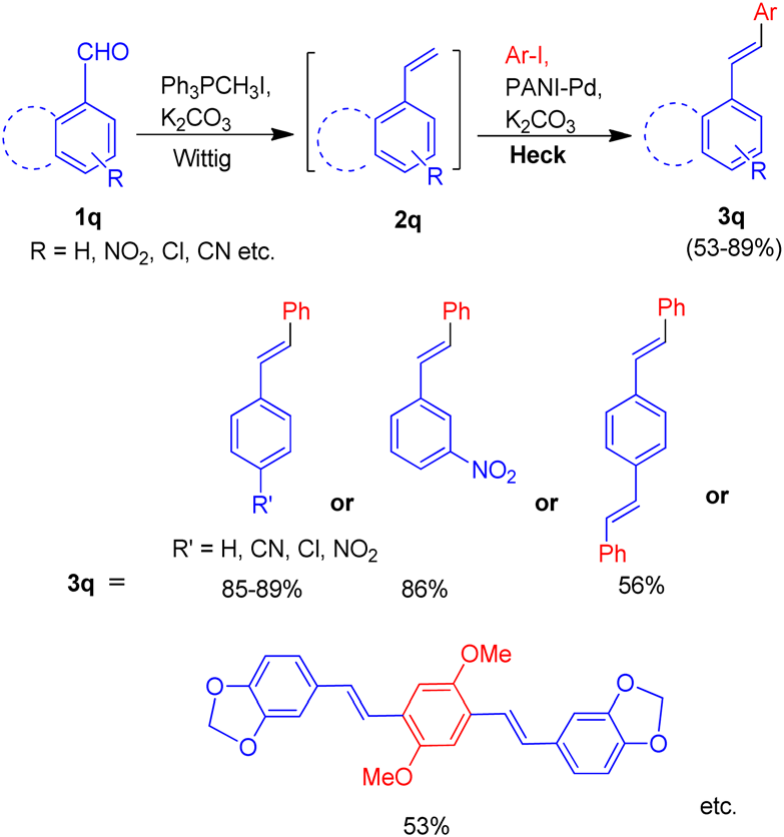
Substrate scope for one-pot Wittig–Heck reaction catalyzed by PANI-Pd.

In 2014, a polyaniline anchored palladium catalyst (PANI-Pd, Fig. 5) was developed and used by Arun L. Patel, Ashutosh V. Bedekar *et al.*^15^ in one pot Wittig–Heck reaction for the synthesis of a number of stilbene (3q) derivatives (Scheme 30) with good yields (53–89%). The styrene (2q) generated *in situ* through Wittig reaction of an aromatic aldehyde (1q) and Ph_3_PCH_3_I under basic environment, when subjected to Mizoroki–Heck conditions utilizing PANI-Pd as catalyst was able to give stilbene derivatives (3q) in good to excellent yields (Scheme 30). Since the styrene is generated *in situ*, it immediately couples with an aryl halide in the presence of PANI-Pd catalyst to result in the formation of corresponding substituted olefin. The optimized condition employed an 1.2 equiv. of aromatic aldehyde (1q) with a wittig salt, methyltriphenylphosphonium iodide (1.4 equiv.) at 60 °C for wittig reaction for *in situ* synthesis of substituted styrene (2q) which on providing Mizoroki–Heck coupling conditions (presence of PANI-Pd catalyst (0.02 equiv.) with dry potassium carbonate (3 equiv.) in dry DMA at 140 °C for 40 h) couples with aryl iodide to form corresponding olefin (3q) in good yields (53–89%).

The catalyst *i.e.* PANI-Pd, despite only being applicable for aryl bromides and aryl iodides and not for aryl chlorides, displayed high efficiency and provided a wide range of tolerance for functional groups. The presence of electron withdrawing groups on various aryl halides analyzed does not seem to affect the overall yield of the desired product.

**Scheme 31:**
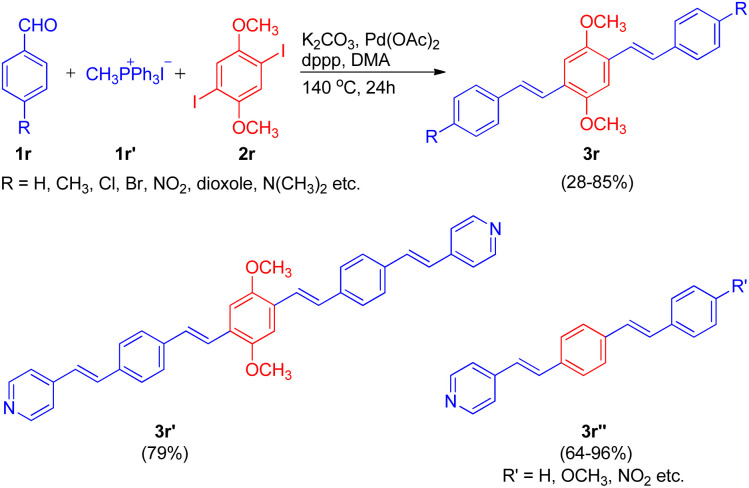
One-pot Wittig–Heck synthetic methodology for the synthesis of OPV's.

Extending their work, in order to synthesize a series of oligo(phenylenevinylenes), carrying different functional groups to study their spectroscopic properties, Ashutosh V. Bedekar and co-worker in 2015 (ref. 22) utilized an almost similar one-pot Wittig–Heck procedure. The one-pot methodology initiates with the *in situ* generation of substituted styrene *via* Wittig reaction of 4-substituted benzaldehydes (1r; 1 equiv.) with methyl triphenylphosphonium iodide (1r′; 1 equiv.). The styrene thus formed then subsequently undergoes Mizoroki–Heck reaction with 1,4-diiodo-2,5-dimethoxybenzene (2r; 5 equiv.) by utilizing 5 mol% Pd(OAc)_2_ as catalyst, K_2_CO_3_ as base (3.5 equiv.) with TBAB (2 equiv.) and dppp (0.04 equiv.) in *N*,*N*-dimethyl acetamide under nitrogen atmosphere at 140 °C for 24 h, to give the corresponding coupling products 2,5-dimethoxy-1,4-distyryl benzene derivatives (3r) in good yields (28–85%). In similar manner, pyridine containing oligo(phenylenevinylene)s (3r′ and 3r′′) bearing different substituents were also successfully synthesized (64–96%). Thus the one-pot Wittig–Heck methodology efficiently synthesized a number of oligo(phenylenevinylene)'s (OPV) derivatives with various functional groups having different electronic properties in moderate to good yield (28–96%) to study their optical properties (Scheme 31).

**Fig. 6:**
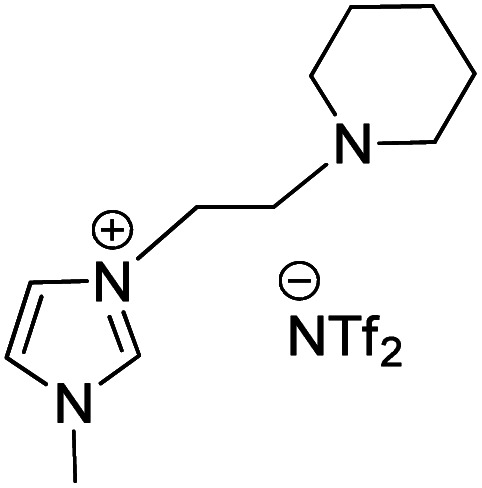
Showing structure of [PAIM][NTf2] ionic liquid.

**Scheme 32:**
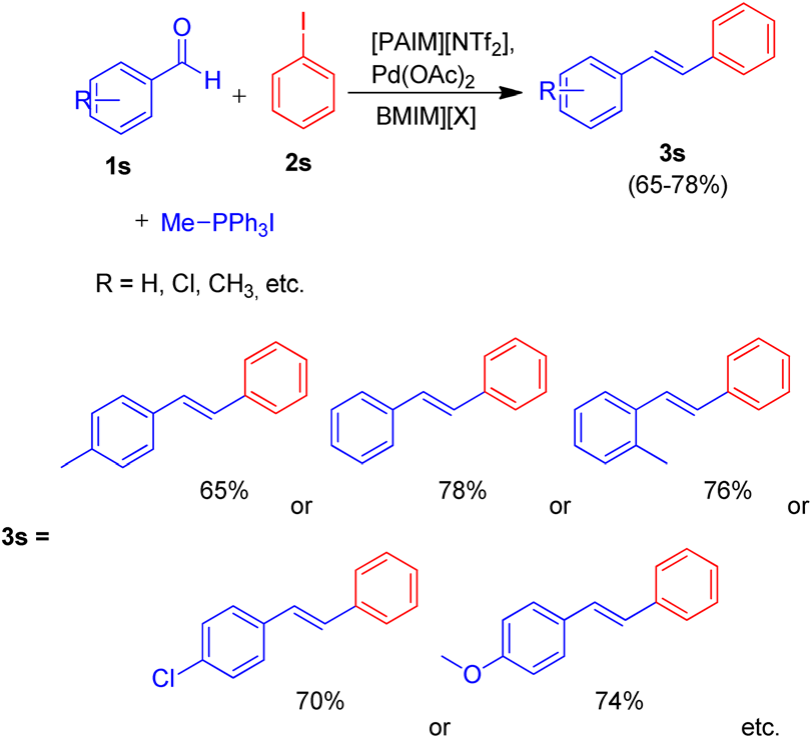
Wittig–Heck tandem reactions for the synthesis of substituted *trans*-diphenyl-ethenes in ionic liquid.

Owing to the various advantages offered by ionic-liquids in palladium-catalyzed cross-coupling reactions, their various classes have been used in Heck reaction. Kenneth K. Laali and co-workers in 2017 (ref. 23) thus utilized the piperidine-appended imidazolium-IL [PAIM][NTf_2_] (Fig. 6), for the synthesis of substituted diarylethenes *via* sequential Wittig–Heck reaction. The process first involved Wittig olefination of benzaldehyde (1s) (by utilizing methyltriphenylphosphonium bromide as Wittig reagent), the olefin thus formed then underwent Mizoroki Heck coupling with PhI (2s) in presence of palladium catalyst in order to form substituted *trans*-diphenyl-ethenes (3s) in good to moderate yield (65–78%) (Scheme 32).

Various aromatic aldehydes were analyzed for the said protocol under optimized condition involving utilization of 1.3 equiv. of aromatic aldehyde, 1.2 equiv. of phosphonium salt as Wittig reagent, 1 equiv. of iodobenzene, in the presence of 3 equiv. of basic ionic liquid (piperidine-appended imidazolium-IL [PAIM][NTf_2_]) and 10 mol% of Pd(OAc)_2_ as catalyst in [BMIM][X] (X = PF_6_ or BF_4_) as solvent in an oil bath at 65 °C for 8 h resulted in moderate to good yield (65–78%) of the substituted stilbene with primarily *E*-isomers. The presence of electron withdrawing or electron donating groups on aromatic aldehyde does not affect the product yield significantly.

This method was able to show the efficiency of ionic liquid for the development of simple one pot Wittig–Heck cross-coupling reaction occurring without the addition of any additive.

The Wittig–Heck reaction was also extended for the synthesis of fluorine-containing s-shaped π-conjugated dibenzo[*c*,*l*]chrysene derivative by Tetsuji Moriguchi and co-workers in 2017 (ref. 24) in order to study it's photophysical properties as well as determine the structure owing to it's importance in semiconducting material and other electronic devices.

**Scheme 33:**
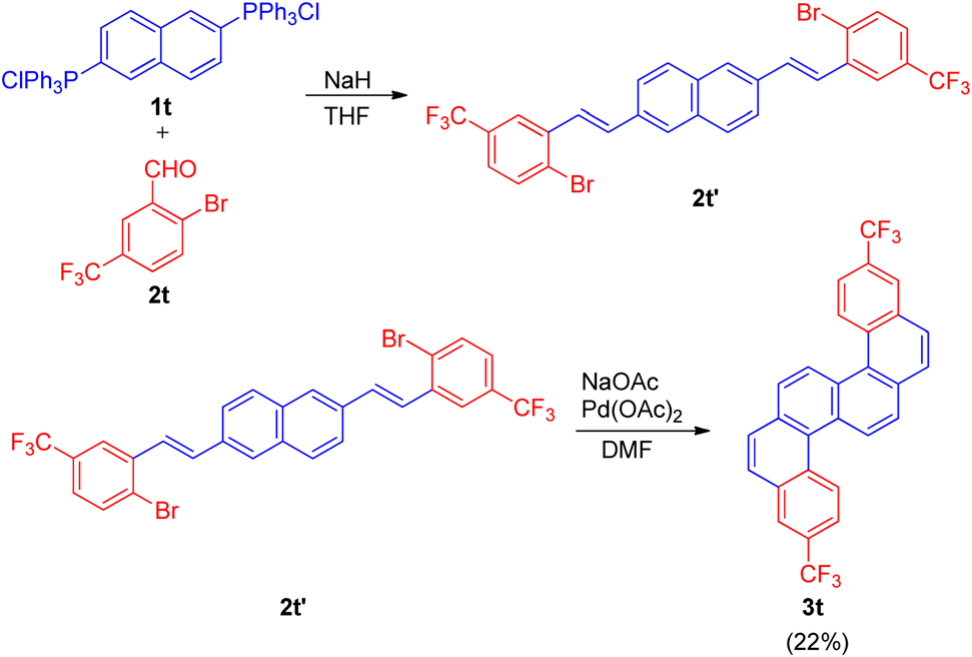
Synthesis of isomeric fluorinated s-shaped polyaromatic dibenzo[*c*,*l*]chrysene derivative (3t).

The s-shaped polyaromatic compound was synthesised in 2-step Wittig–Heck reaction. The 1st step involved condensation of a Wittig-salt (1t; 0.5 equiv.) with trifluoromethyl benzaldehyde (2t; 1 equiv.) *via* Wittig-reaction to generate the desired olefin (2t′) which then in the 2nd step undergoes an intramolecular Heck reaction in presence of Pd(OAc)_2_ (5 mol%) as catalyst, NaOAc (5 equiv.) as base in DMF at 110 °C for 18 h to result in the formation of desired substituted and highly conjugated cyclic system (3t) however with low yield of 22% (Scheme 33).

**Scheme 34:**
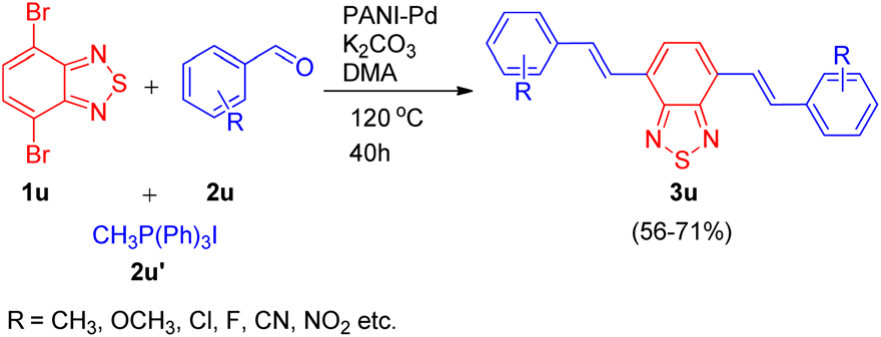
PANI-Pd catalyzed one-pot Wittig–Heck reaction for the synthesis of benzochalcogendiazole-based conjugated molecules.

Utilizing the same catalyst developed by Arun L. Patel, Ashutosh V. Bedekar *et al.*^15^ in 2014, Arun L. Patel and co-workers in 2019,^25^ developed an efficient synthetic approach for formation of benzochalcogendiazole based π-conjugated molecules in order to study their photophysical properties. The path leading to the formation of a new class of substituted benzothiazole derivatives exploited the same one-pot Wittig–Heck reaction^11^ as given by them in 2014. The method involved *in situ* generation of vinyl arenes *via* reaction of an aldehyde (2u; 1 equiv.) and Ph_3_PCH_3_I (2u′; 2.2 equiv.), which when subjected to Mizoroki–Heck conditions, *i.e.* on providing a nitrogen atmosphere at 120 °C for 40 h and utilizing potassium carbonate as base (12 equiv.), and PANI-Pd as catalyst (12 mol%) in DMA, couples with dibromobenzothiadiazole (1u; 1 eq.), resulting in formation of bis(vinylarene)-capped benzochalcogendiazoles (3u; Scheme 34). When a series of electron rich and electron poor benzaldehydes were made to react with phosphonium salt to generate the required olefin for undergoing heck coupling with 1u, varied number of conjugated fluorescent heck products (3u) were synthesized in good yields (56–71%).

**Scheme 35:**
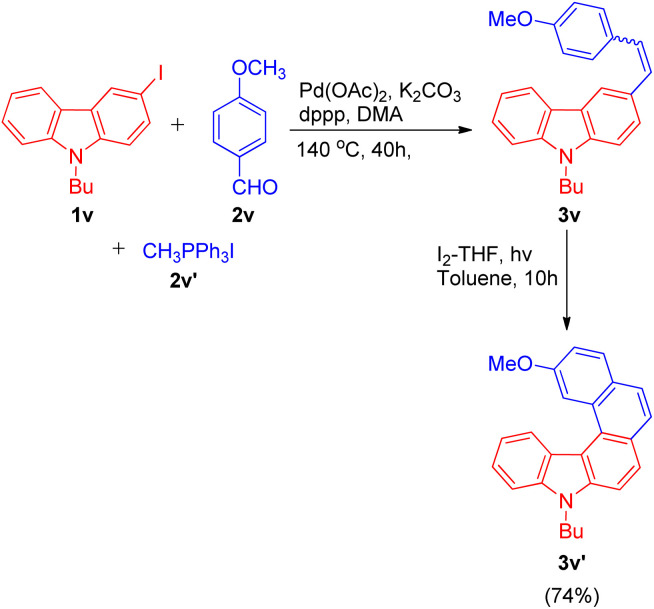
Synthesis of aza[5]helicene.

The one-pot Wittig–Heck reaction given by Ashutosh V. Bedekar was also utilized again in 2021 by Ashutosh V. Bedekar and co-workers^26^ as one of the key steps for the synthesis of aza[5]helicenes derivatives to study their photophysical properties and effect of methyl substituent in fjord region. Apart from being an important moiety in the area of material science, aza[5]helicenes derivatives also act as an efficient model for regioselectivity, configurational stability, and side reactions, illustrations. The reaction begins with *in situ* generation of required alkene 3v (4-methoxystyrene) by the Wittig reaction of *p*-anisaldehyde (2v; 1.5 equiv.), and methyl triphenylphosphonium iodide (2v′; 1.5 equiv.). The alkene thus formed reacts subsequently with halogen substituted 9*H*-carbazole (1v; 1 equiv.) on providing Heck reaction conditions *i.e.* using 5 equiv. K_2_CO_3_ a base, 0.2 mol% Pd(OAc)_2_ as catalyst, with 0.02 equiv. dppp and 0.2 equiv. TBAB in *N*,*N*-dimethyl acetamide under nitrogen atmosphere at 140 °C for 24 h to result in formation of corresponding carbazole compound 3v (Scheme 35). Further photochemical cyclization of the resulting alkene in presence of I_2_ and THF lead to the formation of desired cyclized aza[5]helicene (3v′) in 74% yield.

The aza[5]helicene (3v′) thus generated was investigated further for it's photophysical properties and other structural parameters to gain an insight into the effect of methyl substituent onto their conformation stability in the fjord region.

**Scheme 36:**
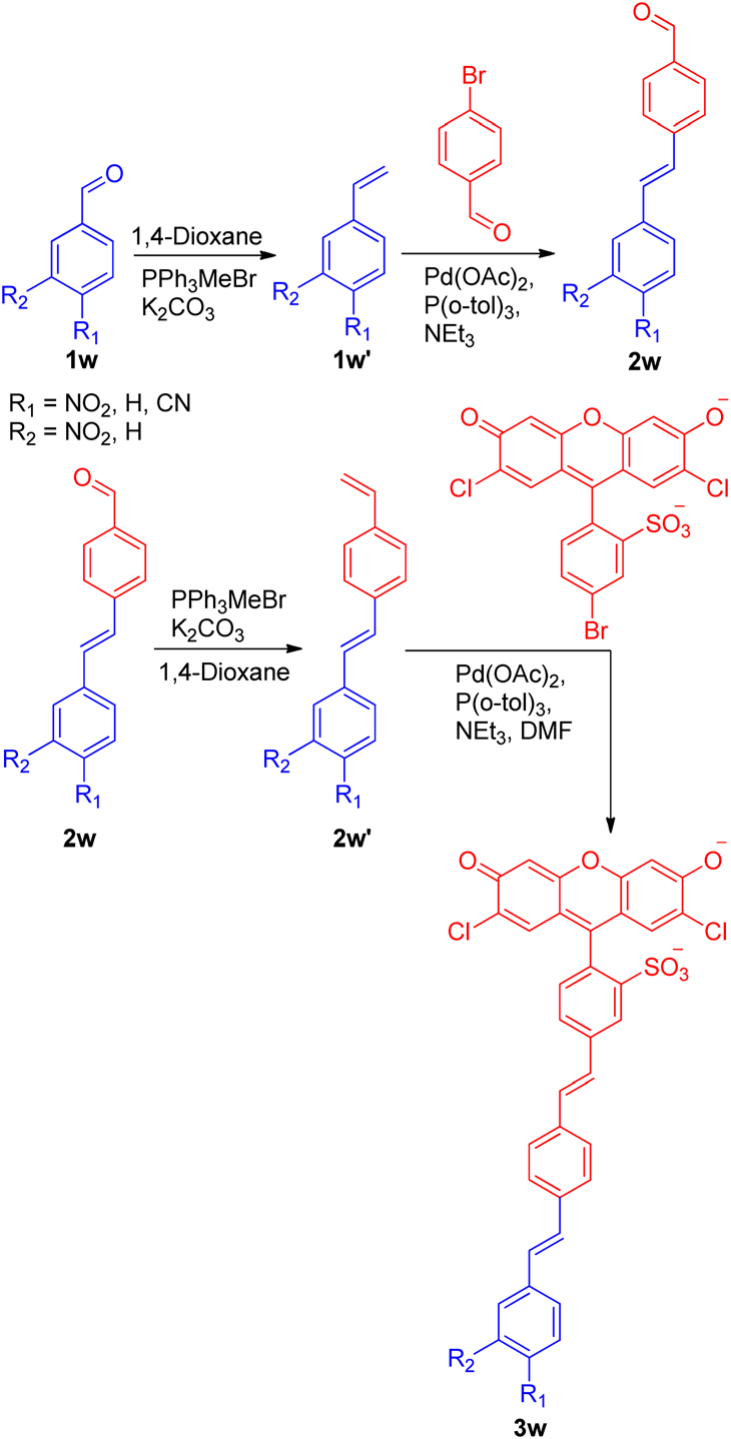
Synthesis routes of 4-NO_2_-VF, 3-NO_2_-VF, and 4-CN-VF.

A recent application of the Wittig–Heck pathway was seen in a successful attempt to synthesize and study the reverse demand voltage fluorophores by Evan W. Miller and co-workers in 2022.^27^ The development of 3 out of 5 voltage-sensitive fluorophores (VF dyes) exploited Wittig–Heck reaction as a key step. Out of five, three electron withdrawing groups (4-NO_2_-, 3-NO_2_-, and 4-CN-) containing substituted stilbenes were synthesized *via* domino Wittig/Heck/Wittig reaction carried out using Pd(OAc)_2_ as catalyst, K_2_CO_3_ as base in 1,4-dioxane as solvent giving the desired VF dyes (Scheme 36) to study their response to membrane potential. Among the three VF dye developed using Wittig–Heck reaction, 4-NO_2_-VF (3w) was observed to be voltage sensitive.

## 4. Conclusion

Thus summarizing from the above discussion, the difficulties encountered upon direct usage of alkenes during Mizoroki–Heck coupling such as their purification and manufacturing *etc.*, lead to the invention of *in situ* generation of desired alkenes through various means and then utilizing them in Heck coupling. For the Mizoroki–Heck reaction these alternative ways for generating alkenes apart from employing Wittig reaction, involves hydrolysis followed by dehydrohalogenation, simple dehydrohalogenation, dehydration, elimination or deacetoxylation of various alkenes synthons. However the synthesis of olefins, required for Heck coupling through Wittig reaction has become more appealing in recent years due to substantially greater availability of aromatic aldehyde and ketones as compared to styrene.^15^

These reactions not only did solve the issue of alkenes undergoing polymerization when stored or reacted at high temperature but the one-pot Wittig–Heck sequence also significantly increased the reaction efficiency by reducing the reaction time and purification steps.^20^ Thus this alternative route to *in situ* generation of alkenes followed by their coupling with aryl halides to form substituted olefins must be explored further in future owing to their myriad of applications.

## Conflicts of interest

There are no conflicts to declare.

## Supplementary Material
